# Requirements for Portable Instrument Suites during Human Scientific Exploration of Mars

**DOI:** 10.1089/ast.2018.1841

**Published:** 2019-03-06

**Authors:** Alexander Sehlke, Zara Mirmalek, David Burtt, Christopher W. Haberle, Delia Santiago-Materese, Shannon E. Kobs Nawotniak, Scott S. Hughes, W. Brent Garry, Nathan Bramall, Adrian J. Brown, Jennifer L. Heldmann, Darlene S.S. Lim

**Affiliations:** ^1^ NASA Ames Research Center, Moffett Field, California.; ^2^ Kennedy School of Government, Harvard University, Cambridge, Massachusetts.; ^3^ BAER Institute, Moffett Field, California.; ^4^ Department of Geosciences, Stony Brook University, Stony Brook, New York.; ^5^ Mars Space Flight Facility, Arizona State University, Tempe, Arizona.; ^6^ Deparment of Geosciences, Idaho State University, Pocatello, Idaho.; ^7^ NASA Goddard Space Flight Center, Greenbelt, Maryland.; ^8^ Leiden Measurement Technology LLC, Sunnyvale, California.; ^9^ NASA Headquarters, Washington, District of Columbia.

**Keywords:** Handheld spectrometers, Exploration, Operations, Basalt, Extra-vehicular activities, Mars

## Abstract

Human explorers on the surface of Mars will have access to a far wider array of scientific tools than previous crewed planetary exploration missions, but not every tool will be compatible with the restrictions of this exploration. Spectrometers on flyby, orbital, and landed missions are currently used to determine the composition and mineralogy of geological materials of various types and sizes, from small fragments to celestial bodies in the solar system. Handheld spectrometers that are capable of *in situ* analyses are already used for geological exploration on Earth; however, their usefulness for human exploration missions and how data from multiple handheld instruments could be combined to enhance scientific return must be further evaluated. As part of the Biologic Analog Science Associated with Lava Terrains (BASALT) research project, we incorporated two handheld instruments, a visible-near infrared spectrometer and an X-Ray Fluorescence spectrometer, into simulated Mars exploration missions conducted on basaltic terrains in Idaho and Hawai'i. To understand the data quality provided by these handheld spectrometers, we evaluated their performance under varying conditions of measurement time, distance, angle, atmosphere, and sample matrix, and we compared data quality between handheld instruments and laboratory techniques. Here, we summarize these findings, provide guidelines and requirements on how to effectively incorporate these instruments into human exploration missions to Mars, and posit that future iterations of these instruments will be beneficial for enhancing science returned from human exploration missions.

## 1. Introduction

NASA's Journey to Mars will require a paradigm shift in our operational and engineering designs to support humans as they conduct science and exploration in the realm of deep space and beyond. NASA analog research activities offer scientific and operational approximations of lunar- and Mars-like environments where innovations can be safely and efficiently developed, tested, and applied in preparation for future missions (Todd and Reagan, 2003 [NEEMO]; Lim *et al.*, 2011 [PLRP]; Ross *et al.*, 2013 [DRATS]). Although humans cannot yet themselves voyage to Mars to test and develop tools and protocols related to sample selection, we can use analog environments and the framework of associated research projects to flesh out scientific, technical, and design requirements in service of this end goal.

Within the realm of these analog missions, research programs are able to address instrument design pertaining to the development of these systems (*e.g.*, handheld spectrometers and analytical tools) for future space flight (Young *et al.*, 2013). These programs leverage both available commercial off-the-shelf (COTS) technologies and the instrument heritage of past and current NASA robotic exploration missions during testing and informing design requirements.

Crewed missions designed to characterize the surfaces and environments of other terrestrial bodies in our solar system will need to incorporate flight instruments that provide rapid *in situ* evaluation of planetary landscapes. Specialized analytical tool suites will be critical to the high grading and triage of samples during extra-vehicular activities (EVAs), in which crews on Mars and Earth use the information provided by these analytical tools to decide which locations to sample. Efficient and accurate instruments will enhance scientific return during high-risk human excursions onto the martian surface.

Spectroscopy, a powerful and often nondestructive method, is used to study the mineralogy and geochemical composition of rocky bodies in our solar system, by flybys, orbiters, or landers (*e.g.*, Bibring *et al.*, 2006; Head *et al.*, 2008; Ehlmann *et al.*, 2009; Ehlmann and Edwards, 2014; Williford, 2018). Traditionally, on Earth, rock samples are collected in the field and, subsequently, analyzed for high-quality data in a laboratory with instruments that are too large, delicate, and complex to take into the field. The terrestrial process of sample collection and analysis, however, must be re-evaluated for Mars (and the Moon) with the incorporation of portable instruments (*e.g.*, Bishop *et al.*, 2011; Brown *et al.*, 2014) that were not available during the Apollo missions. Time and resources will be limited for the crews, and the imposition of communication latencies between Earth and Mars brings additional levels of complexity to the mission that are not experienced on Apollo (Beaton *et al.*, 2019; Lim *et al.*, 2019; Stevens *et al.*, 2019).

Issues for robotic Mars Sample Return mission design tend to focus on the collection mechanisms (rover-based and/or lander-based sampling systems), and on obtaining the sufficient geological context (color panoramic imaging, micro imaging, mineralogy, elemental abundances) for the samples. After samples are collected, the robotic mission must provide ways to preserve their key characteristics during the return to Earth (Beaty *et al.*, 2008 [iMARS Working Group Final Report]). These issues are transferable and relatable to science-driven human mission plans, particularly as they pertain to EVAs: Crewmembers exploring the surface of Mars (extra-vehicular [EV] crew) will have to balance limited EVA time with decision making that will likely involve an organizational network of “clients” including the intra-vehicular (IV) crew on Mars and an Earth-based science support team (see Lim *et al.*, 2019 for details). As such, transmitting the most diagnostic and informative data back to any of the EVA clients will require both capabilities and Concepts of Operations (ConOps) optimized for the exploration conditions and planned mission architectures.

The ability to distinguish the “best” or the “better” samples to collect in service of science or engineering goals will endure as a component of exploration activities for astronauts on Mars, as it does for all field scientists on Earth who must select wisely from an array of options. To stay within the limits on the total mass of samples that can be returned to Earth (Williford, 2018), the systematic identification of functional requirements for future *in situ* sample high-grading techniques will provide the foundation from which future capabilities (*e.g.*, handheld instruments, data visualization) and operational concepts (*e.g.*, decision protocols, task timing, and planning) will be designed. Ultimately, these requirements will affect not only the scientific success and efficiency but also the overall mission success given that poor instrument design has the potential to lead to frustration and costly inefficiencies.

The focus of our study is the identification of functional requirements associated with *in situ* analysis of basalt targets on celestial bodies, particularly Mars. Basalts are the dominant type of volcanic rocks composing the surface of Earth and other terrestrial bodies in the solar system, including Mars. Since their emplacement millions to billions of years ago (Carr and Head, 2009), martian basalts have been subject to alteration, either during their emplacement or afterward by chemical and mechanical weathering. Basalts vary in the modal and size distributions of crystals and vesicles, as well as exhibit differences in chemical composition. All of these factors make basalts and their alteration products the primary targets for this study, providing implications for the search for microbial life on the martian surface (Cockell *et al.*, 2018).

In this article, we present the results of an investigation on the utility of selected handheld spectrometers within the framework of a simulated Mars mission, namely the Biologic Analog Science Associated with Lava Terrains (BASALT) mission architecture (see Lim *et al.*, 2019 for details). The analog field sites for BASALT are representative of the environmental history of Mars. These sites, located in Idaho and Hawai'i (Hughes *et al.*, 2019), were selected to understand the habitability of terrestrial volcanic terrains as analog environments for early and present-day Mars. The analogue for early Mars is active volcanism at Mauna Ulu and the summit region of Kilauea volcano, Big Island (Hawai'i). The analogue for present-day Mars is the physically and chemically weathered volcanic terrain of Craters of the Moon National Monument and Preserve (Idaho). Fieldwork was conducted by BASALT's EV/IV and science support teams under simulated Mars mission constraints, including 5- and 15-min one-way time-delay (latency), during three deployments (2016–2017).

Two spectrometers were selected based on their prospective ability to enable the BASALT team to acquire high-grade samples in service of their science objectives (see BASALT STM in Lim *et al.*, 2019) while operating under simulated Mars mission conditions in the field. The science objectives required sampling unaltered and altered basalt rock to capture end-member geochemical differences that could result in understanding the influence of alteration types on microbial communities. Field geologists primarily rely on visual changes in physical properties of rocks (*e.g.*, color, texture, morphology) to identify samples that may have a different composition or may have been weathered. The EV crew on another planet will similarly use visual cues to identify samples to collect. The evaluation of the usefulness of the different handheld spectrometers was guided by geological research questions, including: (1) Could differences in degree of alteration be effectively assessed through visual cues? (2) How would each instrument best support visual cues that identify alteration?

### 1.1. BASALT Mars mission architectures and integration of handheld spectrometers

**FIG. 1.**
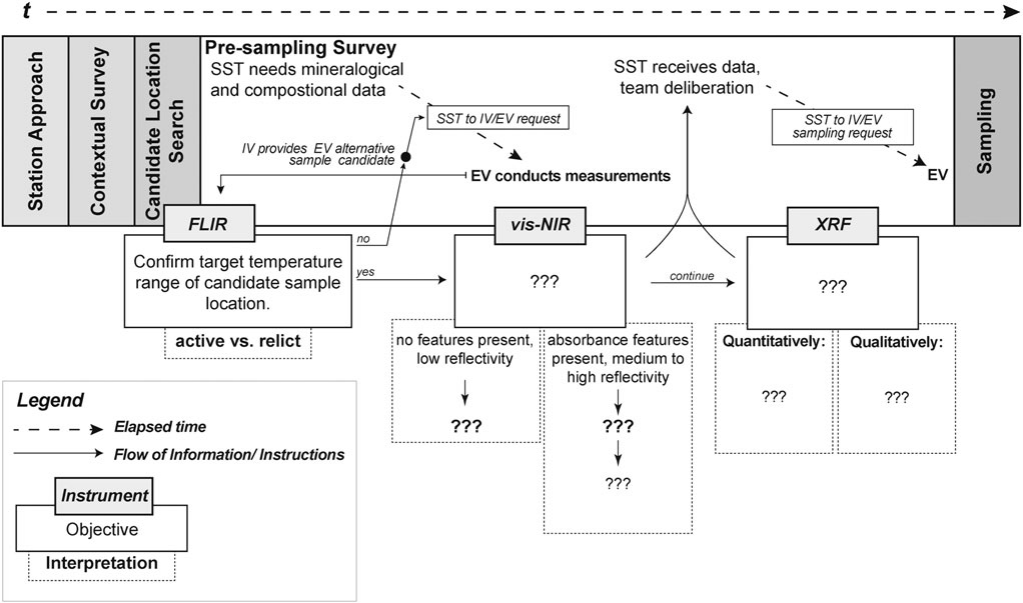
This is an exploratory representation of instrument integration within EVA architecture during phase four, the presampling survey. The notations “???” represent the open questions on specific objectives for which the instrument was potentially useful. At the end of this research paper, we present this same figure updated with our findings. Note: The EVA included the use of an FLIR camera during candidate location search and the presampling survey. Even though its usefulness was not evaluated in our study, we decided to reference the FLIR to be consistent with other studies describing the EVA of the BASALT research program. BASALT, Biologic Analog Science Associated with Lava Terrains; EVA, extra-vehicular activities; FLIR, forward-looking infrared.

BASALT EVAs were divided into five main phases, executed in the following chronological order: (1) station approach, (2) contextual survey, (3) sample location search, (4) presampling survey, and (5) sampling (Fig. 1). The approximate length of an EVA was 4 to 6 h. During EVA phases, a team of scientists (Science Support Team, SST) in a Mission Support Center (MSC) communicated with EV crew via IV crew to receive data and provide sample prioritization recommendations (for details, see Beaton *et al.*, 2019).

Handheld spectrometers (vis-NIR and XRF) were only used during phase four, the presampling survey, when the previously determined sample locations were further characterized in terms of their mineralogical and chemical components. The EV crew collected spectroscopic data during the presampling survey, whereas the Science Support Team. would review data and deliberate to reach a set of sampling priority recommendations, which would, subsequently, be sent to the IV and EV crew for the sampling phase. The activity time allowed for the presampling survey phase was budgeted for 30 min, by ConOps design. Within this timeframe, two instruments were used for determining the mineralogy and elemental abundances of basaltic rocks and their alteration products. Then, acquired data had to be transmitted easily and quickly for team deliberation among the SST, resulting in recommendations sent to IV crew on site, who act as guides and a communication channel between the EV and SST team, for phase five “sampling.”

Because time is limited during EVAs, it is essential to include technology that provides efficient data acquisition. Handheld spectrometers, which were not available during the Apollo missions, offer the opportunity to enhance these EVAs by providing rapid, *in situ* characterization of rocks (Young *et al.*, 2016, 2018). With the help of these instruments, EV, IV, and SST crew are better positioned to decide on sampling priorities. However, for human exploration missions, the operation (user interface) and data output from the COTS instruments need to be fully understood to maximize their use, optimize their design, and avoid any false or misleading interpretations due to measurement conditions (as discussed in Section 4.4).

### 1.2. Methods and tools used to characterize rocks

Identifying the chemistry and mineralogy of rocks requires a variety of information types, from which geologists can deduce the rocks' origin and the magmatic history of specific geological regions or even global association.

Traditional analytical techniques were developed in laboratory settings, where many are still used in standard practice. For example, methods often include: (1) X-ray diffraction to identify minerals and mineral groups, (2) reflectance and transmission spectroscopy ranging from ultra-violet (UV) up to the mid-infrared (MIR) to identify minerals and certain chemical species (*e.g.*, OH and H_2_O) within these materials (Gurgurewicz *et al.*, 2015), and (3) X-ray fluorescence (XRF) or mass spectrometers to provide high-quality chemical analyses and even isotopic signatures. All of these standard laboratory methods provide high-quality data with low detection limits and high precision. In contrast, adapting these techniques to much smaller, handheld spectrometers may incur loss of resolution, of precision, and general data quality.

One complicating factor of utilizing handheld spectrometers for field measurements is that sample preparation in the field can only be minimal. High-quality data produced by laboratory measurements rely on careful sample preparation (*e.g.*, cutting, polishing, grinding, sieving). Moreover, laboratory measurements, unlike those done in the field, are usually conducted under optimized conditions (*e.g.*, atmospheric controls or vacuum, a temperature-regulated laboratory, etc.).

Interpretation of data returned from handheld instruments must include an understanding of (1) the intrinsic properties of the rock itself, such as variations of the matrix (*e.g.*, knowledge of modal abundances, size of crystals, and vesicles); (2) changes in atmospheric conditions over hours or days (*e.g.*, humidity, temperature, and solar radiation); and (3) outcrop accessibility. All of these occur during field work on Earth, and they will also occur exploring the surface of Mars. Identifying, understanding, and developing protocols for mitigating some or all of the complicating factors is necessary for effective exploration on the surface of other worlds.

We cannot predict where technology will be in 20 years, but we can contribute to a foundation of requirements for technology development generated through analog research and testing. The BASALT research informs an exploratory representation of an instrument suite deployment, with contextual details of team communication and activity times, during an EVA.

In addition to the two geological questions as noted in the Introduction, future human exploration missions need to build on questions that focus on human-technology relationships such as those that guided our assessment of instrument use by EV/IV and science teams in Mars mission conditions, specifically: (1) How does a handheld scientific instrument serve the EVA sample prioritization process both in the field and in the science support team?; (2) What are the most critical conditions—analytical measurement and data transmission—influencing data quality?; (3) Given the scientific objectives, what type and how many instruments are optimal during EVAs?; and finally (4) How can instrument use be synchronized to leverage relative strengths of multiple tools?

## 2. Methods

### 2.1. Suite of portable spectrometers and specifications

**FIG. 2.**
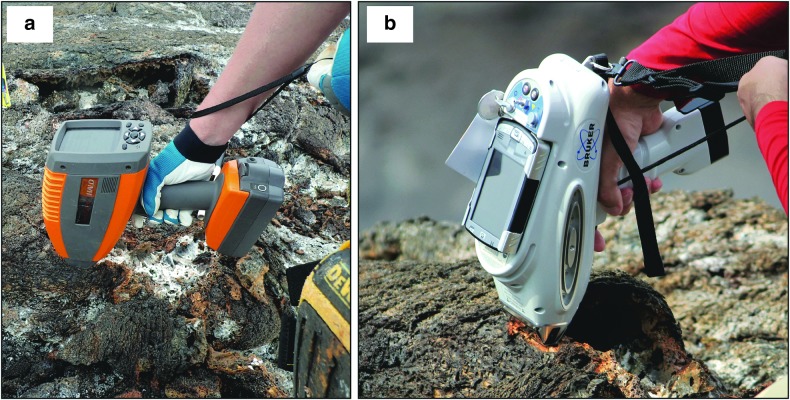
Photographs of both handheld spectrometers in use to high-grade samples during simulated EVAs at Hawai'i Volcanoes National Park: **(a)** vis-NIR, **(b)** XRF. (Photos: Z. Mirmalek).

The two instruments (Fig. 2) used for high-grade candidate samples are a visible-near infrared (vis-NIR) spectrometer, which provides identification of minerals, and an XRF spectrometer, which provides elemental composition of the target. Both techniques are commonly used to characterize geological samples, from which the mineralogy and geochemistry can be derived, allowing interpretations regarding the origin, geological history, and scientific importance of the sample. In this study, we focus on evaluating the following two handheld spectrometers in greater detail: the ASD TerraSpec Halo^®^ portable vis-NIR spectrometer and the Bruker Tracer IV-SD^®^ XRF spectrometer.

The ASD TerraSpec Halo (vis-NIR) is designed to be a portable mineral identifier. The instrument captures continuous single reflectance spectra in the vis-NIR wavelength range (350–2500 nm). Within that spectral range, igneous and alteration mineral groups can be identified (*e.g.*, hydrated and hydroxylated phases, such as phyllosilicates, zeolites, goethite, and sulfates, based on the presence of 1400 and 1900 nm absorption bands; Fe^2+^ bearing phases, such as pyroxene and olivine; and Fe^3+^ bearing phases, such as hematite and magnetite, based on several absorption bands between 400 and 1100 nm).

Samples are illuminated with an internal quartz tungsten halogen bulb with a spot size of ∼18 mm in diameter, whereas the instrument is in contact with the sample. Spectral resolution is 3 nm for wavelengths of 350–700 nm, 9.8 nm for 700–1400 nm, and 8.1 nm for 1400–2100 nm. Depending on the setting (bright or dark surface), the instrument measures 50 (bright) to 100 (dark) spectra in ∼15 s, and it displays an average continuous reflectance spectrum on completion. The spectrometer provides a qualitative assessment of identified minerals using an internal mineral library and fitting routine, including a rating algorithm to indicate interpretation confidence. However, different minerals have similar absorption features; therefore, automatic mineral identification algorithms are most likely limited to identify mineral groups (*e.g.*, zeolites, phyllosilicates) rather than distinguish between specific minerals within a group.

The Bruker Tracer-IV SD XRF spectrometer is capable of producing an anode current of up to 60 μA at 40 kV. The X-ray beam is produced by an Rh target thin window, and secondary electrons are counted by a 10-mm^2^ XFlash^®^ silicon drift detector. Both the X-ray tube and the detector are behind a gridded ∼10-μm-thick prolene window to prevent contamination of these components. An internal filter wheel allows optimization for specialized measurement conditions; however, because the SST was primarily interested in changes of major elements across outcrops, filters were not used.

Our default operating conditions were an anode voltage of 40 kV while applying a 44 μA probe current. The instrument can be connected to a vacuum pump, or flushed with helium (after removing the prolene window) to obtain higher count rates, enabling a more precise quantification of lighter elements, such as Na and Mg. However, the addition of the vacuum pump and/or helium bottle are difficult to integrate into our simulated Mars exploration mission. The spot size is ∼3 mm in diameter, equating to a total excitation area of ∼7 mm^2^. The instrument is operated via an external PDA that displays calibrated element concentrations. Allotted time per individual analysis was set to 30 s as default in our field studies, which is slightly longer than the analyses time for the vis-NIR spectrometer.

### 2.2. Measurement variables and data quality tests

Both intrinsic sample variables and intrinsic instrument variables have a significant impact on data quality and need to be considered in any evaluation of the instruments. These variables are detailed as follows.

#### 2.2.1. Intrinsic sample variables

As previously outlined, the geological samples in our exploration missions are basaltic rock. Therefore, they exhibit uneven surfaces and have variable textures, including varying porosity and distributions of vesicles even within the same hand sample, causing a deviation from the ideal configuration where the instrument is normal to, and flush with, the sample surface. Deviation from this ideal setup causes the instrument beam to travel farther distances, which increases interactions with the atmosphere and scattering of photons within the sample itself, which weakens the signal during XRF analyses (*e.g.*, Young *et al.*, 2016). Thus, the data quality for chemically equivalent samples can vary greatly from one sample to another based on surface properties.

#### 2.2.2. Intrinsic instrument variables

The ASD Halo vis-NIR spectrometer is not customizable. The only variable that may be changed is the scan setting between light- and dark-colored rocks, whereby the light setting combines 50 scans over the entire spectrum, and the dark setting averages 100 scans. By contrast, the portable XRF is highly customizable, allowing the user to adjust measurement time, atmospheric controls within the instrument around the detector, and the beam intensity, all of which directly influence the count rate and spectral quality (*e.g.*, overall signal to noise ratio). Hence, the ability to adjust these parameters to optimize measurements for a specific task, such as increasing light-element (Z < Al) sensitivity by increasing the measurement time and/or low-pressure environment around the detector (by attaching a portable vacuum pump), may improve the analysis quality significantly, therefore providing a more robust assessment of the sample during EVAs. Table 1 lists the intrinsic sample and instrument variables investigated in the laboratory to assess their influence on spectral quality, and the detailed description of each test is provided later.

**Table 1.** Variables Tested to Assess Data Quality Influence for vis-Near Infrared and X-Ray-Fluorescence Spectrometer

*Variable tested*	*vis-NIR*	*XRF*
Instrument-sample surface variables		
Distance	Yes	Yes
Angle of incidence	Yes	Yes
Instrument variables		
Analysis time of individual measurement	n.a.	Yes
Atmosphere: pressure at 10^5^ and 10^3^ Pa	n.a.	Yes
Atmosphere: ambient light contamination	Yes	n.a.

n.a., not applicable; vis-NIR, visible-near infrared; XRF, X-ray-fluorescence.

#### 2.2.3. Distance test

**FIG. 3.**
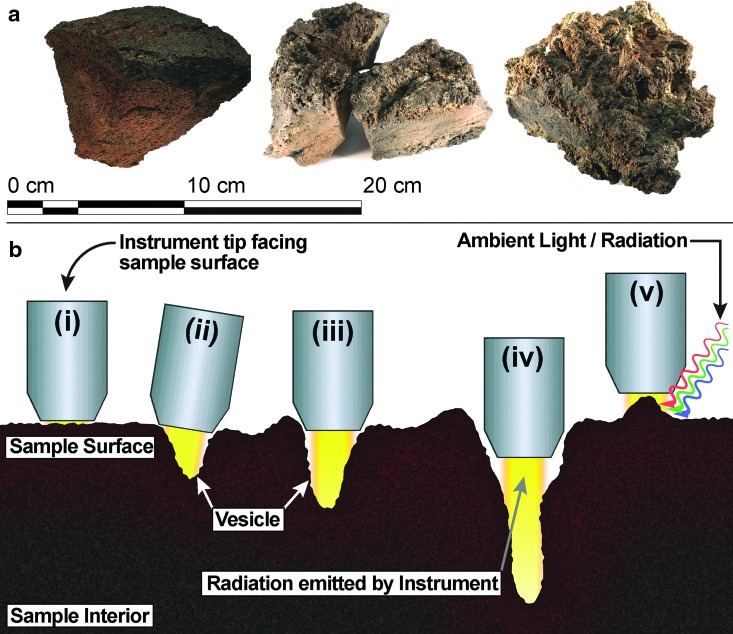
**(a)** Photographs of basaltic rocks that show increasing surface roughness from left to right. **(b)** Schematic representation of holding a contact-based handheld spectrometer against the rock surface that is completely flat (i) and effectively in perfect contact to the sample surface. (ii–iv) Increasing travel distance of radiation emitted by the instrument on uneven surface and void space (vesicles, cracks); (v) contamination with ambient light/radiation measuring the surface of a highpoint while the distance of sample-instrument increases simultaneously.

Uneven surfaces and different vesicle sizes in basalt effectively increase the distance between sample surface and detector (indicated in Fig. 3). To simulate the increased travel distance of photons, we measured the spectra for both spectrometers by stepwise increasing the distance between instrument and sample surface from 0 mm (in contact) up to 20 mm in 2-mm increments. For this test, the instrument was positioned orthogonally to a flat cut sample surface. The sample surface itself was unpolished to remain within similar surface sample conditions encountered in the field.

#### 2.2.4. Angle test

To evaluate the influence of the angle at which the instrument is held against the sample (which corresponds to oblique surfaces or vesicle walls), we stepwise-varied the angular position of the instrument to the flat sample in 5° increments from normal up to 15° to 20° in one direction, which provides a 40° cone of the angle of incidence.

#### 2.2.5. Analysis time and pressure test

This test is only applicable to the XRF, which allows customization of the measurement duration. Generally, the longer the measurement time, the better the signal-to-noise ratio, hence a better spectral quality with increasing measurement time. The quality of the spectra was evaluated for analysis times of 30, 60, 300, 900, and 1800 s. Both time series were conducted at ambient pressure conditions (10^5^ Pa), and a weak vacuum of ∼10^3^ Pa was achieved by attaching a vacuum pump to the instrument. The low-pressure environment mimics ambient pressure conditions on Mars, ignoring the exact compositional differences (N_2_- vs. CO_2_-dominated atmosphere), and it also provides insights into the working conditions in other low-pressure environments or airless bodies in the Solar System.

We measured five spots on each sample in our sample suite (see description of samples used in Section 2.3) at each time and pressure condition while the instrument was in contact with the flat sample surface. A new measurement location was chosen after the time and pressure series was completed. For example, we measured one spot at atmospheric conditions between 30 and 1800 s; then, we connected the vacuum pump and measured the time series at 10^3^ Pa for the same spot. After this was completed, a new measurement location was chosen. This measurement protocol allowed for direct comparison of the data quality between time and pressure conditions (10^5^ vs. 10^3^ Pa). The assessment of the data quality was then expressed as the root-mean-square standard deviation (RMSD) between the externally determined geochemistry data (*e.g.*, mass spectrometry, microprobe) and the values predicted by the instrument.

#### 2.2.6. Light contamination test

This test is only applicable to the vis-NIR instrument. Here, we conducted the distance test (see above) with four different lighting conditions: (1) in direct sunlight at mid-day, (2) in a fully shaded area during sunlight, (3) in a room illuminated by fluorescent light bulbs, and (4) under red light (photographic darkroom). The goal was to detect the influence of ambient light contaminating the spectrum, mimicking light conditions experienced in the field, and the distance of instrument to sample surface at which ambient light would contaminate the instrument spectrum. No light flux/quality measurements were conducted to quantify the lighting conditions. However, we can roughly estimate the lighting conditions based on historic values of daily solar radiation measurements and computations for inclined surfaces up to 15° (uncertainty of the sample alignment) for latitudes of 40 degrees northern hemisphere (∼ San Jose, CA) between April and May, which averages around 342 ± 14 W m^−2^ (Buffon *et al.*, 1972).

### 2.3. Sample suite for instrument assessment in the laboratory

**FIG. 4.**
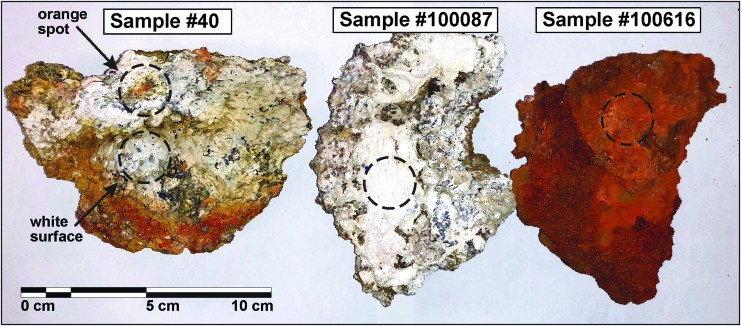
Photographs of the samples from Kilauea used for the distance and angle test with vis-NIR spectrometer. Black dashed circles mark the locations and extent of the measurement spots for the vis-NIR distance tests at different lighting conditions. Measurement surfaces are fairly flat and unpolished surfaces.

Three samples collected during the Hawai'i 2016 deployment at Mauna Ulu, Kīlauea volcano, Hawai'i, were used to evaluate the vis-NIR spectrometer: (1) a highly altered, low-density basalt (Sample No. 40) collected at an active fumarole at Kane Nui O Hamo, (2) a sample from an active fumarole at Mauna Ulu (No. 100087), and (3) one syn-emplacement sample (No. 100616) shown in Figure 4. Sample surfaces on which the vis-NIR spectrometer was tested were fairly flat, unpolished surfaces. These samples were collected to test the instrument against different colored surfaces to check whether or not the quality of the spectra with increasing distance and angle of incidence are affected similarly for brighter/darker surfaces (*e.g.*, white versus dark red).

The standards used to calibrate and assess our handheld XRF unit range in composition from basalt to basaltic andesite, all of which are unaltered (Table 2). The largest portion of these XRF samples are from the Eastern Snake River Plain, namely Craters of the Moon (COM) Kings Bowl Pit (KBP), and Inferno Chasm (IC). One sample was erupted at Mauna Ulu (MU), Kīlauea volcano, Hawai'i, and one at Pacaya (PA) volcano, Guatemala.

**Table 2.** X-Ray-Fluorescence Calibration Standards of Known Geochemistry

	*SiO_2_*	*TiO_2_*	*Al_2_O_3_*	*FeO*	*MnO*	*MgO*	*CaO*	*Na_2_O*	*K_2_O*	*P_2_O_5_*	*Total*
Terrestrial rock samples											
14-PA-17^a^	50.55	1.16	18.72	10.16	0.18	4.38	9.49	3.32	0.88	1.16	100.00
COM-14-01	50.33	2.89	13.11	15.90	0.29	3.21	7.00	3.25	2.13	1.88	100.00
COM-14-02	50.36	2.87	13.17	15.89	0.29	3.18	7.00	3.24	2.13	1.86	100.00
COM-14-04	63.21	0.74	14.37	9.08	0.19	0.32	3.15	4.12	4.64	0.18	100.00
COM-14-05	64.10	0.64	14.26	8.58	0.19	0.21	2.96	4.08	4.82	0.14	100.00
COM-14-06	64.13	0.65	14.21	8.64	0.19	0.22	2.96	4.03	4.83	0.14	100.00
COM-14-07	63.02	0.75	14.41	9.15	0.19	0.34	3.20	4.15	4.58	0.19	100.00
IC-14-01	47.78	2.03	15.29	11.93	0.20	8.61	10.96	2.24	0.48	0.48	100.00
IC-14-05	47.64	2.05	15.20	12.05	0.20	8.61	11.23	2.15	0.42	0.46	100.00
KBP-14-01	48.43	2.19	15.70	11.91	0.20	6.88	11.27	2.40	0.55	0.47	100.00
KBP-14-02	48.61	2.26	15.45	11.97	0.20	6.69	11.32	2.46	0.57	0.47	100.00
KBP-14-03	48.61	2.28	15.51	12.08	0.20	6.64	11.28	2.38	0.56	0.46	100.00
MU-6^b^	49.79	2.14	12.14	11.67	0.17	11.52	9.98	1.96	0.41	0.21	100.00
Planetary analog glasses^c^											
COM-BD	49.77	2.95	13.18	15.93	0.30	3.21	7.14	3.41	2.13	1.98	100.00
EUC	48.67	0.68	13.00	18.16	0.20	7.31	11.17	0.48	0.12	0.22	100.00
IcP-HCT	53.70	0.90	12.40	3.34	0.23	22.70	6.34	0.16	0.19	0.04	100.00
KOM	50.35	0.37	10.62	11.07	0.11	17.27	9.59	0.44	0.14	0.04	100.00
KREEPe	53.31	2.34	15.42	7.66	0.16	8.23	10.92	0.95	0.91	0.09	100.00
LM	42.91	9.71	8.78	19.18	0.10	5.82	12.90	0.35	0.18	0.06	100.00
NAK	48.08	0.76	12.49	21.39	0.42	4.10	10.15	2.21	0.10	0.29	100.00
NVP-Na	55.88	0.91	15.12	2.95	0.25	13.84	4.37	6.45	0.22	0.00	100.00
NVP-Nae	58.58	1.11	19.11	2.65	0.11	5.77	5.30	7.08	0.27	0.01	100.00

Bulk composition from ^a^Soldati *et al.* (2016), ^b^Robert *et al.* (2014) and Sehlke *et al.* (2014), and ^c^Sehlke and Whittington (2016).

Measurements were done on flat cut (but unpolished) surfaces. These specimens provide surfaces of the unaltered rock, composed of its igneous texture (crystals embedded in a glassy matrix). In addition to these natural rocks, we used planetary analog glasses (Sehlke and Whittington, 2016) to cover an even wider range in chemical compositions. Some of these planetary glasses are extremely different in composition from terrestrial basalts/lavas, so their inclusion provided a good opportunity to test how well a calibration capable of covering great variety in major elements can be developed. In total, 22 samples were used for calibration and quality assessment, providing a wide chemical range to calibrate the instrument.

### 2.4. vis-NIR field-based performance associated with varying basaltic alteration products

To understand how to optimize the use of ASD-type data products during Mars EVA operations, we performed a series of field-based performance assessments on a basalt flow lobe with surface color gradients hypothesized to derive from variability in alteration products.

For example, black surfaces are consistent with unaltered basalts, composed of a mixture of primary minerals (olivine, pyroxene, feldspar, spinel) in a glassy matrix. In contrast, we define altered basalts as basaltic rock that has undergone chemical, mineralogical, and textural changes, usually exhibiting red coloration as a result of Fe-oxidation; orange and tan colors due to abundance of Fe-bearing clay minerals, white to tan colors for zeolites, and bright yellow colors to (elemental) sulfur deposits.

These experiments were focused on understanding how the ASD spectral capabilities would best serve our science team's interest in identifying unaltered and highly altered basalts, particularly under the Mars EVA conditions that inherently imposed specific sample high-grading constraints on our field team. It is important to underscore that we were not assessing the broader intrinsic value of the ASD instrument to scientific field work.

**FIG. 5.**
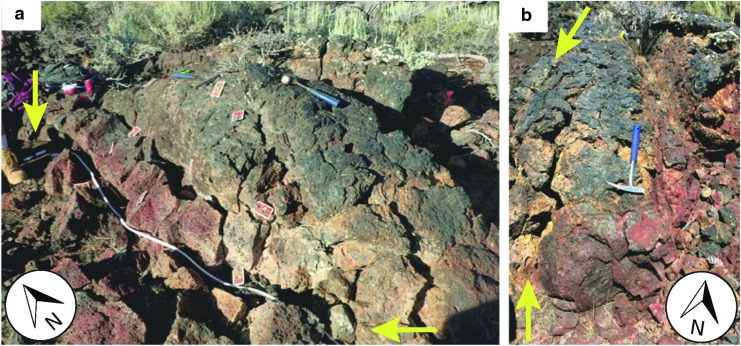
The outcrop at the Big Craters region of Craters of the Moon National Monument and Preserve in Idaho. **(a)** Sampling was performed with the ASD instrument along a 10-cm grid beginning at the proximal end (upper left corner of image), numbered 1, to the distal end (bottom right corner), numbered 22. The bottom of the lobe was assigned the letter A, leading up to letter J at the top of the lobe. **(b)** The distal end of the lobe (rock hammer has been repositioned between images). Yellow arrows indicate length/width of the horizontal transect. Rock hammer length is ∼30 cm. (Photos: D.S.S. Lim).

The field site of interest was located in the Big Craters region of Craters of the Moon National Monument and Preserve, Idaho. The outcrop, resembling a flow lobe within a larger basalt flow, measured about 2.5 m in length, 1.0 m in height, and 1.8 m in width, and it included a surface color gradient that progressed from dark brown-black at the proximal end to red and yellow at the distal (terminal) end of the lobe (Fig. 5). A 10-cm grid was established across the surface of the west-facing side of the lobe. This grid allowed for systematic sampling across the color gradient associated with the lobe surface. At each 10-cm interval, three replicate ASD scans were acquired in the same spot to assess the reproducibility and consistency of mineral data output associated with the color gradient.

The raw data collected for the lobe are in Supplementary Table S1 (Supplementary data are available online at https://www.liebertpub.com/suppl/doi/10.1089/ast.2018.1841), detailing the color designations (visualized in Supplementary Fig. S1) that our team associated with the lobe (Fig. 3) and possible alteration mineral matches (including “no match”) provided by the ASD spectrometer (note: mineral library included alteration minerals). These data were synthesized into three categories that detailed whether a specific mineral output was a primary result that was either 1, 2, or 3 times during replicate scans. During these (replicated) scans, the instrument was always held in contact with the surface at the exact same spot, assuring that the triplicate measurements are directly comparable.

### 2.5. Measurements during simulated EVAs

The presampling survey during the EVA has a nominal length of 30 min, during which time the handheld spectrometers are used to gather data on outcrops and characterize whether or not these outcrops fulfill the objectives of the particular EVA. Therefore, the total time that the instruments can be used is limited. Because the vis-NIR spectrometer is not customizable in any way other than the scan setting for either dark- or light-colored rock, this equates to settings that are either longer or shorter measurement times, respectively.

Since most of the basaltic rocks and their alteration products are dark, we kept the scan setting to “dark rock,” such that each measurement required about 15 s. The XRF has more options to customize its measurement time; however, preliminary tests and the manufacturer's recommendations led us to use 30 s for measurements in the field. We did not consider attaching a vacuum pump or helium tank to the instrument during the EVA, as we tried to minimize the load carried into the field. Due to the low atmospheric pressure on Mars, measurement conditions would be improved on the martian surface. Filters were not used to enhance detection of certain elements at the expense of losing detection of other elements. On average, 15 vis-NIR and XRF spectra were collected, totaling ∼13 min of measurement time per EVA.

The goals of the field tests were (1) to evaluate the best working conditions for the instruments and their operational pitfalls, (2) to evaluate whether the initial EVA measurement conditions were appropriate, (3) to recommend adjustments to the EVA measurement conditions as necessary, and (4) to evaluate the applicability of these technologies for interpreting degree of alteration in Mars- and Moon-relevant basaltic environments.

## 3. Results

### 3.1. vis-NIR assessment

#### 3.1.1. Distance test

**FIG. 6.**
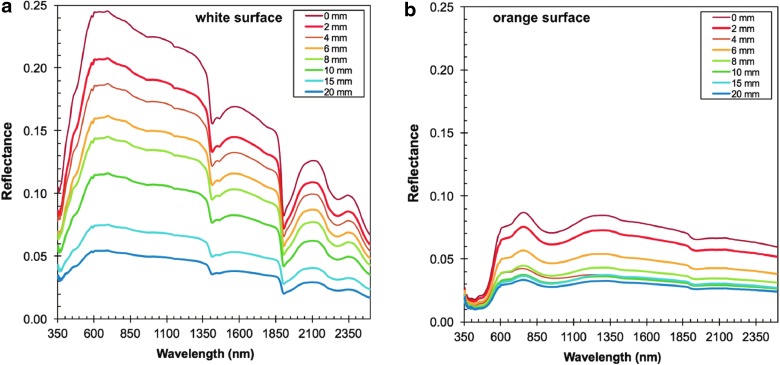
Vis–NIR distance test. Reflectance measurement with the vis-NIR instrument from 0 mm (direct sample contact) up to a 20-mm distance for a vesicular basalt recovered from an active fumarole (Sample No. 40) at a white **(a)** and orange **(b)** surface. Major absorption features are present even at greater distances of 20 mm; however, smaller absorption features in the 600 to 750 nm, and 1400 nm region almost disappear. Influence of atmospheric light is detected at distances greater than 10 mm. Uncertainty of instrument-sample surface distance is ±1 mm.

Figure 6 shows the measured reflectance for white (a) and orange (b) surfaces on the vesicular basalt (Sample No. 40) with increasing distance in red light (dark room). Reflectance decreases ∼50% within the first ∼8 mm. Though major absorption bands from hydrated phases (*e.g.*, OH and H_2_O overtone and combination bands at ∼1.4 and ∼1.9 μm) are present in all spectra, their intensity decreases with increasing distance. The same is observed for minor absorption features (*e.g.*, Fe^3+^ between ∼0.4 and ∼1.0 μm). The automatic mineral identification provided by the instrument software/algorithm is consistent between 2 and 10 mm; with larger distances, some minerals were no longer recognized.

EVA activities occur during daylight, at least in our simulated Mars missions, so we checked whether ambient light might interfere with the instrument data. To do this, we conducted distance tests during mid-day both in total shade (under a tree) and in direct sunlight.

**FIG. 7.**
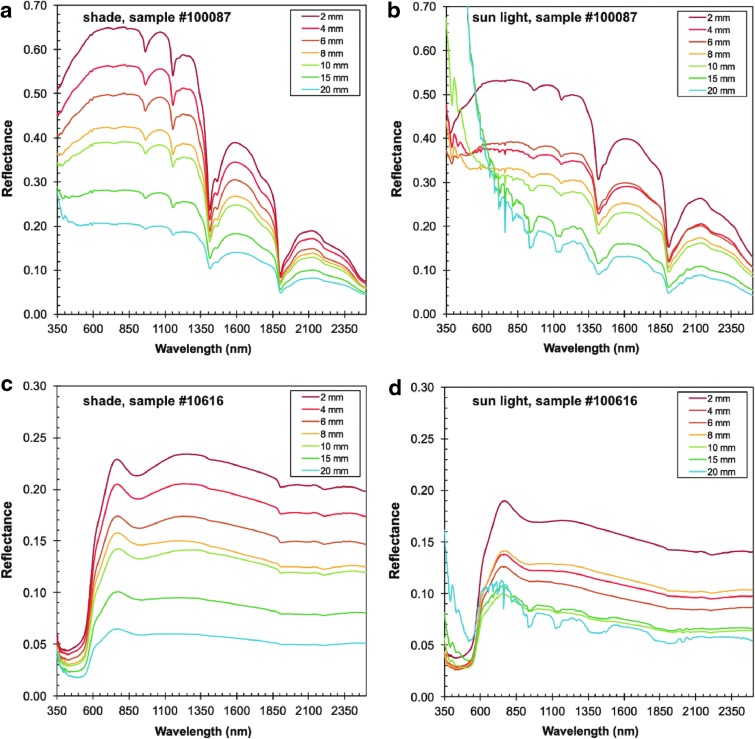
Vis-NIR distance test (shade vs. sun) for sample #100087 **(a, b)** and #100616 **(c, d)** shown in Figure 4. Comparison of reflectance measurements done outside in the shade **(a, c** in left panel**)** and measurements in direct sunlight **(b, d** in right panel**)** with increasing distance. Atmospheric light contamination is observed for the shade **(a, c)** conditions in the spectral range between 350 and 550 nm at distances greater than 15 mm. In direct sunlight **(b, d** in right panel**)**, the entire spectrum is contaminated with ambient light at gap distances of only 10 mm.

Our results are plotted in Figure 7 for two representative samples. At distances of 10 mm and more, atmospheric light contaminates our reflectance spectra, and therefore yields spurious data. Conversely, at distances below 10 mm, no appreciable effect of ambient light contamination was observed. In general, the atmospheric light contamination is more apparent in direct sunlight than in shade, whereby only the 350- to 550-nm region appears to be influenced in the shade, whereas the entire spectrum (350–2500 nm) has a strong solar overprint.

In summary, gaps between instrument and sample surface larger than 10 mm should be avoided for measurements on sunlit surfaces. We also recommend to equip/enclose the instrument tip and sample surface with shielding material to block direct illumination by foreign light sources (other than the instruments' light source) to reduce the amount of ambient light.

#### 3.1.2. Angle test

**FIG. 8.**
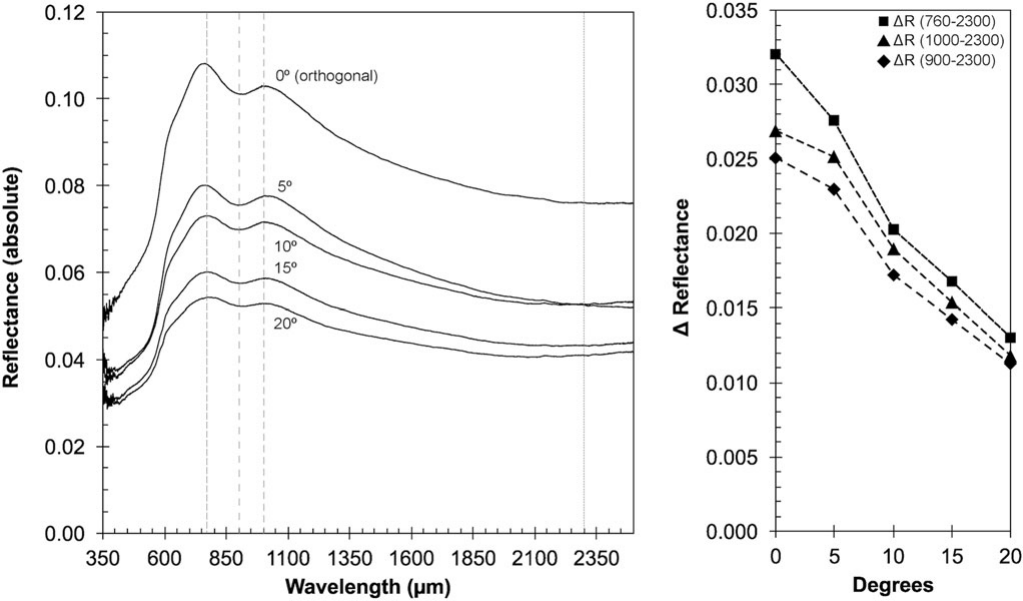
ASD angle test. Changes in absolute reflectance (left) depending on the angle of incidence when the instrument is in contact with the sample. The right portion of the diagram shows the difference (Δ) between an arbitrary baseline at 2300 nm to the absolute reflectance for spectral features at 760, 900, and 1000 nm (faint dashed lines), showing that absolute reflectance decreases about 50% within 20 degrees from orthogonal position of the instrument to the sample surface.

As the vis-NIR instrument is relatively large compared with the test spot size, it can be difficult at some rock outcrops to place the instrument normal to and flush against the surface. Our angle test shows that the reflectivity is uniformly reduced by ∼50% and band depths decrease significantly with up to a 20-degree angle of incidence. This affects spectral quality less than increasing distance from the sample. As such, instrument inclination is less of a concern than instrument distance, but reduced band depths could impede the identification of minor minerals in geological samples. Assessment results are visualized in Figure 8.

#### 3.1.3. Automatic mineral identification and its reproducibility

**FIG. 9.**
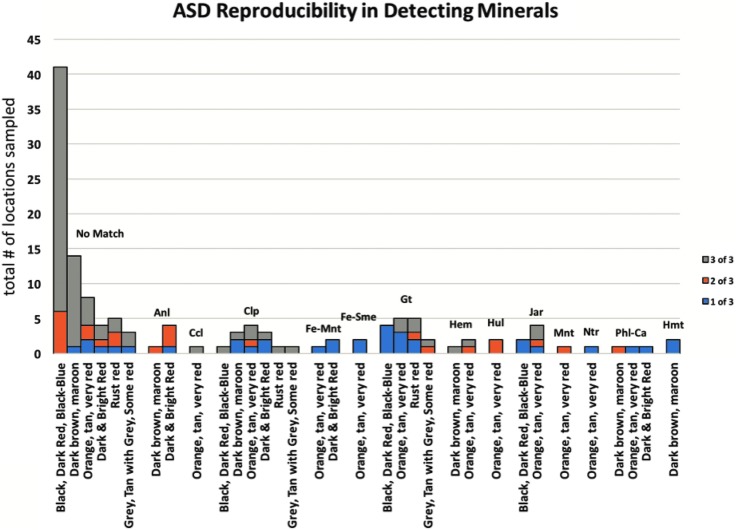
Reproducibility in detecting minerals in various rock colors by the ASD spectrometer. The total number of locations sampled is along the *y*-axis, with different reproducibility values (1, 2, or 3 out of 3 possible attempts) stacked on top of each other and grouped by mineral horizontally. Only scans that appeared as the most confident (3-star rating) mineral identification are included in this figure. For full source data, please see Supplementary Table S1. Minerals are abbreviated as follows: Aln, Analcime; Ccl, Chrysocolla; Clp, Clinoptilolite; Fe-Mnt, Fe-Montmorillonite; Fe-Sme, Fe-Smectite; Gt, Goethite; Hem, Hematite; Hul, Heulandite; Jar, Jarosite; Mnt, Montmorillonite; Ntr, Nontronite; Phl, Phillipsite with Calcium; Hmt, Harmotome. The full list of mineral abbreviations is given in Supplementary Table S2.

Figure 9 graphically represents output from Supplementary Table S1, displaying mineral identifications provided by the instrument software with the highest amount of confidence (3-star rating) for the basalt outcrop shown in Figure 2. Data for each mineral identified were clustered together and arranged by color gradient/designation along the *x*-axis. For each combination of mineral match and rock color gradient/designation, the total number of sampled locations were stacked/summed in a column in the *y*-direction. Within that column, the relative number of samples that replicated mineral matching results with 1, 2, or 3 scans (out of a total of three scans) are shown.

We found that the darker, less altered basalts ranging in color designation from black to dark brown and maroon were most consistently associated with a “no match” designation by the ASD spectrometer. The dominantly “no match” output by the instrument software for black to maroon-colored surfaces is a consequence of (1) the nature of the sample, meaning that the absence of surficial minerals with strong absorption bands (*e.g.*, Fe^3+^, OH^-^, H_2_O) in the spectral range from 350 to 2500 nm results in relatively flat reflectance spectra to which the mineral identification algorithm is unable to match any absorption features, and (2) due to the mineral library loaded onto the instrument, which includes secondary/alteration minerals only, because the objective of the BASALT field studies is to discern between unaltered and altered basalts.

By contrast, the brighter surface colors (orange, tan, white) resulted in a wider variety of alteration mineral identifications, namely oxides, hydroxides, zeolites, phyllosilicates, and sulfur mineralization. These results are consistent with lab-based findings that the more significantly altered basalts revealed a broader array of mineral types than dark samples that had undergone less alteration of the matrix (primary minerals and glass) to secondary minerals. In summary, visual cues, such as coloration of the rock surface, appear to be an adequate proxy to identify secondary mineralization on the surface of basaltic rocks.

Although the various colored surfaces of basaltic rocks indicate various secondary mineralization, questions remain about the reproducibility of such mineral identification algorithms. In our case, reproducibility is defined as the number of identical readings out of triplicate measurements. For example, black surfaces mostly return three “no match” readings, whereas only a small fraction (∼10%) returned “no match” for two out of a triplicate measurement (Fig. 9). Similarly, a minor fraction of “no match” readings is even given for lighter colored surfaces, such as dark brown to maroon, dark red to orange and tan. The individual mineral readings to the right of “no match” cluster in Figure 5 are found by various surface colorations, whereby 1 and/or 2 readings out of a triplicate measurement are dominated.

To address the issue of these false mineral identifications, we also interpreted the mineralogy based on the location and strength of spectral bands; then, we compared our interpretation with the minerals identified by the software. Although bands were correctly identified by the software, the minerals identified were, in many cases, incorrect. For example, tourmaline was identified by the software; however, the absorption bands between 2.2 and 2.3 microns are more likely attributable to phyllosilicates (*i.e.*, Fe-smectite or Mg-illite). In summary, we find that the auto-identification process for this and perhaps similar instruments are worth improvement and that human interpretation is still required to accurately assess the mineralogy of colored basaltic surfaces.

### 3.2. X-ray fluorescence assessment

#### 3.2.1. Time and atmosphere test

**FIG. 10.**
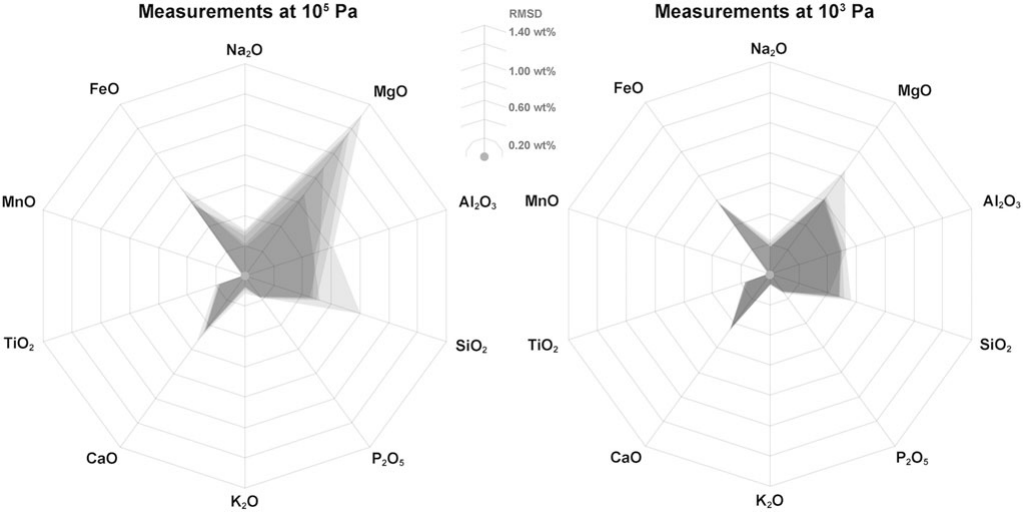
XRF time and atmosphere test. Results of the time and atmosphere test for rock samples only (no glasses included). Quality is expressed as RMSD of 1σ between externally determined bulk chemistry and the predictions based on the portable XRF calibration at 10^5^ Pa (left) and attaching a vacuum pump yielding a pressure of 10^3^ Pa (right). Largest error envelopes (gray) are observed for shortest measurement times (30 s), and they become gradually smaller (gray overlay) with increasing measurement duration (60, 300, 900, 1800 s). The weight percent oxides are arranged clockwise by increasing weight of the metal cation. RMSD, root-mean-square standard deviation.

Figure 10 summarizes the results of the XRF time and atmosphere test, which was measured on cut and polished rock sections. This reflects the ideal conditions, as there is no gap between the rock surface and the instrument nose. With increasing time, the analysis quality expressed as a 1σ RMSD between the external analysis and the prediction of the handheld XRF in weight percent (wt%) is reduced across all major elements (expressed as oxides).

The largest deviations are observed for MgO, Al_2_O_3_, SiO_2_, and FeO. However, since the abundance of SiO_2_ in basalts is high relative to other oxides (*i.e.*, 45–52 wt.% SiO_2_), this only equates to a small relative error and would not result in a misinterpretation of the classification of an igneous rock. MgO and Al_2_O_3_ abundances are lower; therefore, these RMSDs weigh more. The best reproduced oxides are CaO, K_2_O, TiO_2_, and P_2_O_5_. Except for SiO_2_, increasing the analysis time to 60 s provides only small improvements to the data quality, whereby the RMSD is reduced ∼0.08 wt% on average across the entire suite of all major element oxides and only ∼0.03 for the best reproduced oxides.

At lower pressure (10^3^ Pa), the data quality is remarkably increased across all major elements. Measurements at low pressure generally have RMSD that are only half that obtained for the same analysis time at 1 atmosphere. Similar to 10^5^ Pa, data quality is only slightly improved at using integration times above 60 s. Thus, the same suite of elements that is well calibrated under ambient pressure (CaO, K_2_O, TiO_2_, and FeO) is even better calibrated under low-pressure conditions.

In summary, measurement times of 30 s, as employed during the EVA, are sufficient for providing robust geochemical data, although using 60 s integration times and a low-pressure atmosphere around the detector (as it would be encountered on Mars with a pressure of ∼10^3^ Pa) are preferred, if that extra measurement time can be made available during EVAs.

**FIG. 11.**
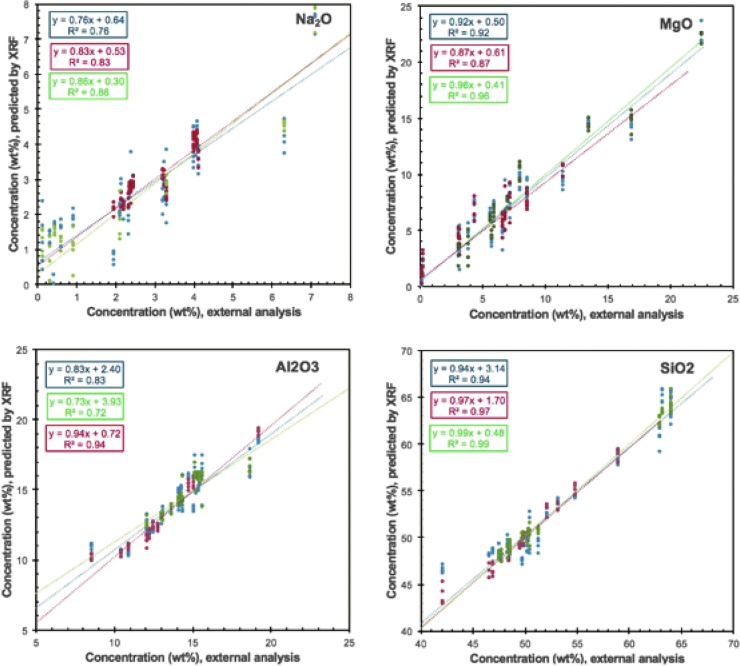

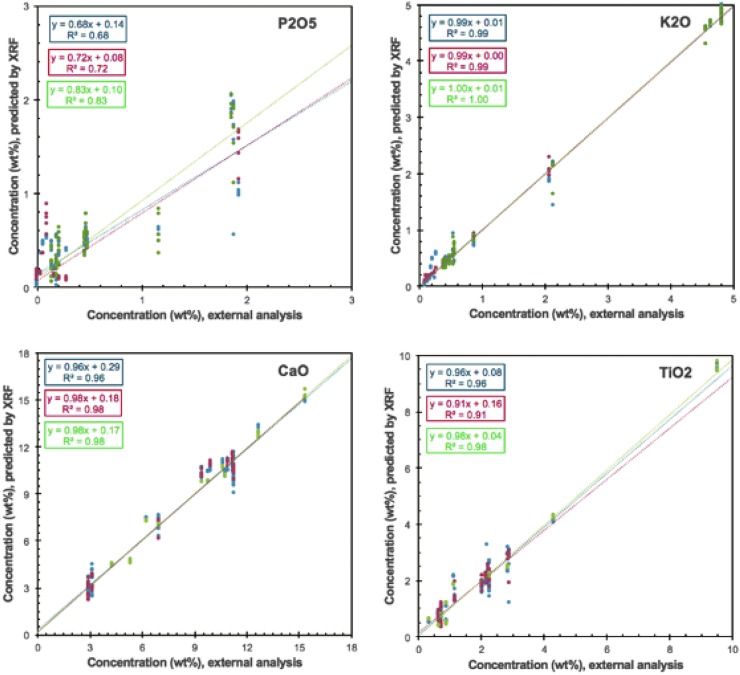
XRF calibration plots with samples of different matrices (partially crystalline rocks and glasses only). Known (externally determined) elemental abundances (expressed as weight percent) are plotted on the *x*-axis, and pXRF predictions are plotted on the *y*-axis. We show a linear correlation of calibrations based on rocks and glass (blue), rocks only (red), and glass samples only (green). Measurement conditions are for 300 s at 10^5^ Pa.

**FIG. 12.**
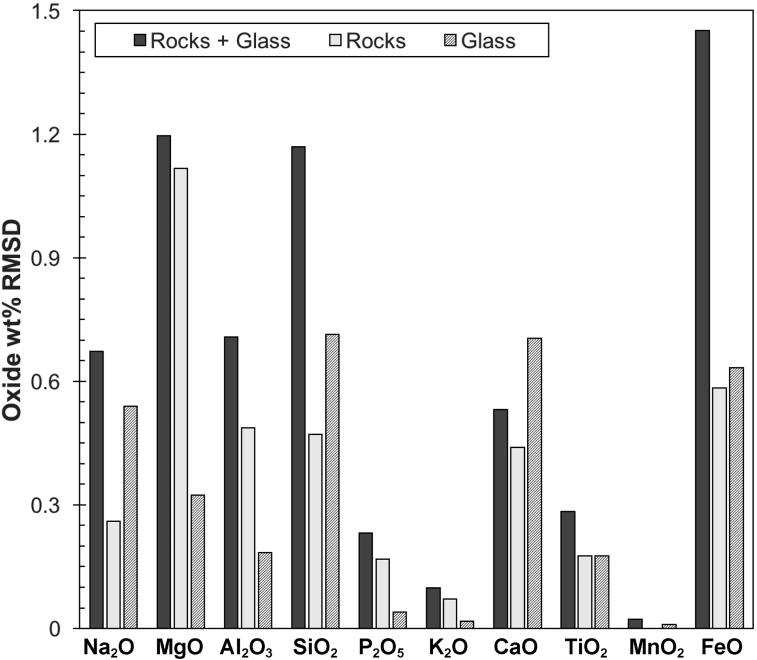
XRF matrix effect. Shown are RMSD of external analysis to the predicted chemical abundances by the Bruker Tracer IV-SD, including rocks and glasses (dark gray), rocks only (light gray), and glasses only (crosshatch) measured at 300 s and 1 atmosphere for each major element oxide. IV, intra-vehicular.

Figure 11 shows the correlation for measurements done at 300 s and 10^5^ Pa by using additional samples to the sample suite described earlier, namely planetary analog glasses. Calibrating the instrument based on rock (crystals embedded in a glass matrix) and glass samples separately provides better linear correlation. Building a calibration based on the entire data set (rocks and glasses) increases the RMSD, presumably due to the very different matrices of both sample types, difficulties in creating a calibration over a wider range of compositional data (introduced by the glasses), or a combination of the two. The changes in RMSD for each oxide using individual calibrations are summarized in Figure 12, suggesting that individual calibrations based on the matrix (glassy vs. crystalline) may yield more accurate quantifications of major elements.

#### 3.2.2. Distance test

Tests of the XRF spectrometer identical to those made with the ASD Halo vis-NIR spectrometer were also conducted to investigate how distance between the instrument to the sample affects data quality. We performed these tests on a martian analog glass representing a Nakhlite composition with ideal conditions. Such a glassy sample has the advantage of being flat and chemically and texturally homogeneous (no crystals or vesicles) to study the effect of data quality with distance alone.

**FIG. 13.**
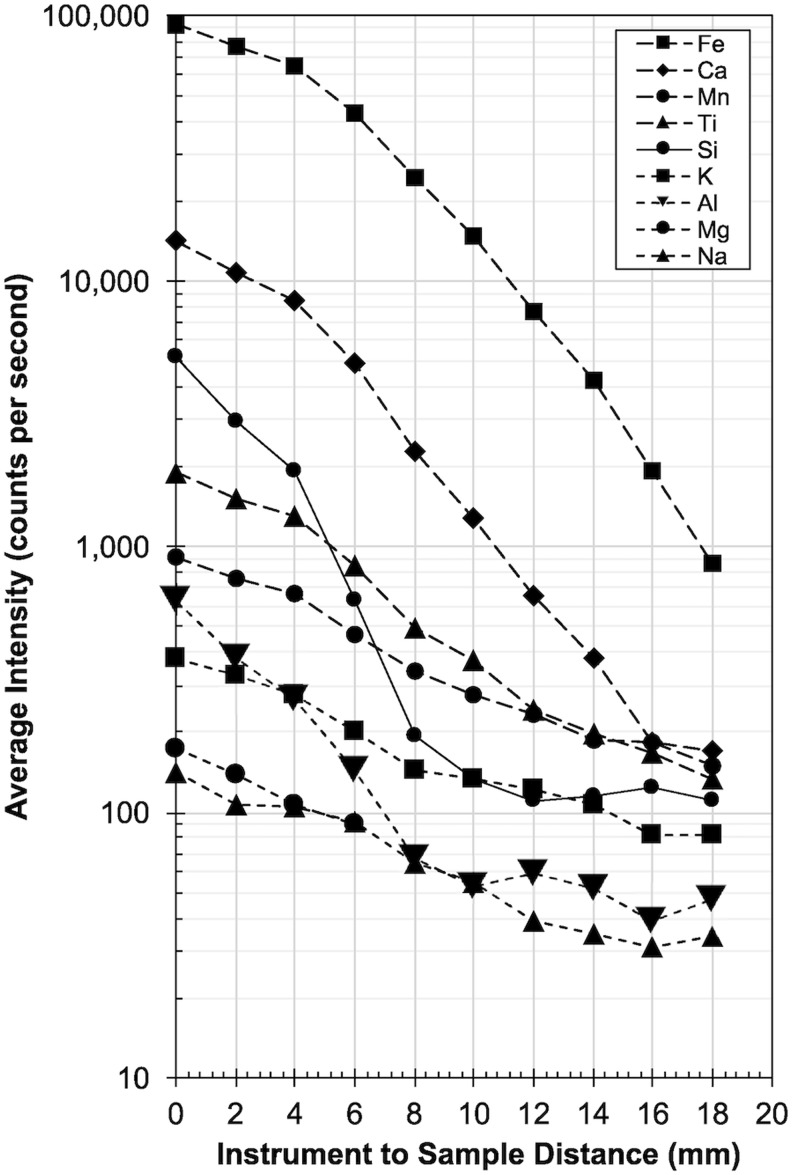
XRF distance test. Plot of the signal strength (in counts for Kα emission lines) versus the distance between the sample and the pXRF to mimic the effects of uneven surfaces. Measurements were taken on a Nakhlite analog glass (see Table 3 for composition) for a duration of 30 s at 10^3^ Pa for every 2 ± 1 mm. With the instrument held at a distance of ∼6 mm from the sample surface, the count rate is only half of that compared with measurements taken directly with the instrument against the sample surface (0 mm).

Our findings are summarized in Figure 13 for measurements at 10^3^ Pa and 30 s. We note that the counts on the detector drop by at least one order of magnitude at distances greater than 10 mm. For example, elements such as Si, Al, and Mg have much different counts when the instrument is in contact or only a few mm off the surface and converge to the same level toward greater distances. This observed loss and convergence of detected X-ray (counts) with distance causes the instrument to report an apparent elemental abundance that is much different than what is real.

Such deviation from the true composition with increasing distance, and how this equates into a shift in reported chemical composition is summarized in Table 3 for a basalt (COM-14-02) at 300 s at 10^5^ and 10^3^ Pa, as well as for the Nakhlite glass at 10^5^ Pa. At distances greater than 4 mm, the composition predicted by the XRF is outside of the standard error (RMSD, discussed earlier).

**Table 3.** Changes in Chemical Composition with Distance for pXRF Measurements

	*SiO_2_*	*TiO_2_*	*Al_2_O_3_*	*FeO*	*MnO*	*MgO*	*CaO*	*Na_2_O*	*K_2_O*	*P_2_O_5_*	*Total*	Total^*^
Distance COM-14-02, 300 s at 10^3^ Pa												
External	50.36	2.87	13.17	15.89	0.29	3.18	7.00	3.24	2.13	1.86	100.00	(100.00)
0 mm	49.40	2.88	13.43	15.15	0.27	5.02	7.57	2.33	2.24	1.71	100.00	(97.15)
2 mm	50.35	2.80	13.82	14.54	0.26	4.74	7.90	1.92	2.24	1.43	100.00	(93.14)
4 mm	53.23	2.29	14.30	12.22	0.23	5.54	7.72	1.85	1.91	0.72	100.00	(90.02)
6 mm	57.19	1.51	14.42	9.52	0.23	7.33	7.22	1.26	1.25	0.07	100.00	(88.92)
8 mm	59.25	0.95	14.86	8.34	0.27	8.57	6.98	n.d.	0.79	n.d.	100.00	(87.64)
Distance COM-14-02, 300 s at 10^3^ Pa												
0 mm	51.83	2.83	12.93	15.06	0.26	3.74	6.64	2.43	2.18	2.11	100.00	(98.74)
2 mm	53.56	2.68	12.82	14.20	0.25	3.64	6.41	2.03	2.17	2.25	100.00	(96.56)
4 mm	54.91	2.44	14.20	13.43	0.25	3.54	6.17	1.57	2.03	1.47	100.00	(86.79)
6 mm	55.60	2.02	17.22	12.29	0.26	3.79	5.86	1.43	1.53	n.d.	100.00	(73.27)
8 mm	56.20	1.45	19.70	10.87	0.31	4.39	5.30	0.85	0.93	n.d.	100.00	(65.69)
Distance Nakhlite (glass), 300 s at 10^5^ Pa												
External	48.08	0.76	12.49	21.39	0.42	4.10	10.15	2.21	0.10	0.29	100.00	(96.92)
0 mm	52.56	0.94	11.58	19.14	0.37	2.37	10.51	2.43	0.03	0.07	100.00	(91.73)
2 mm	50.70	1.07	11.02	18.20	0.35	4.47	10.87	3.13	n.d.	0.19	100.00	(91.38)
4 mm	56.53	1.38	9.89	15.55	0.31	2.71	11.38	1.99	n.d.	0.26	100.00	(85.89)
6 mm	62.49	1.77	9.01	9.09	0.24	1.81	10.92	4.34	n.d.	0.34	100.00	(85.34)
8 mm	62.31	2.15	10.01	4.84	0.22	8.01	10.43	1.47	n.d.	0.56	100.00	(82.26)
10 mm	63.82	2.49	10.76	1.63	0.24	10.21	10.05	n.d.	n.d.	0.80	100.00	(76.85)
12 mm	60.47	2.86	13.30	n.d.	0.25	12.78	9.17	n.d.	n.d.	1.18	100.00	(71.32)
14 mm	51.20	2.97	14.31	n.d.	0.25	22.06	7.75	n.d.	n.d.	1.47	100.00	(71.77)
16 mm	39.70	3.34	18.56	n.d.	0.24	29.87	6.42	n.d.	n.d.	1.87	100.00	(66.47)
18 mm	17.84	3.49	21.39	n.d.	0.19	50.86	4.17	n.d.	n.d.	2.06	100.00	(65.41)

External: Composition provided by independent laboratory analysis (ICP-MS, EMP) for reference. Distance: Gap between instrument and flat surface sample.

^a^ Unnormalized total (raw total) given for context regarding analysis quality.

n.d., not detected.

#### 3.2.3. Angle test

**FIG. 14.**
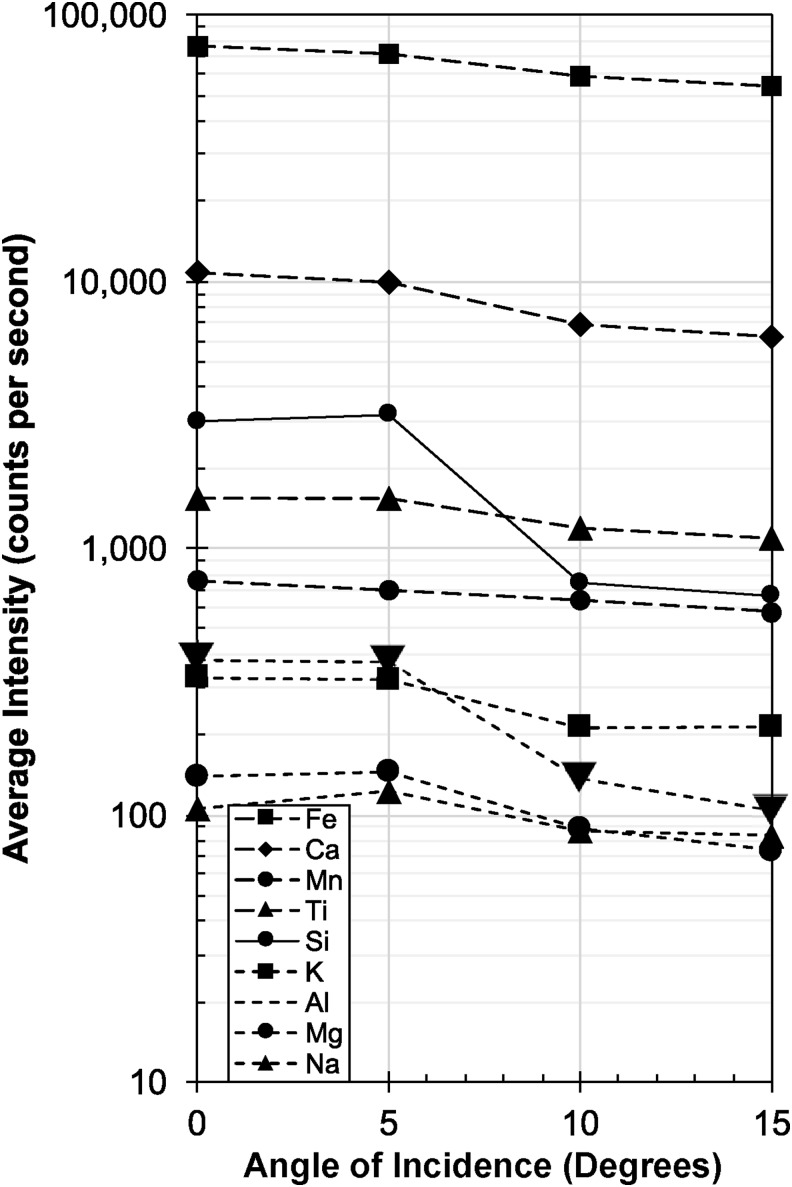
XRF angle test. Plot shows the loss of peak intensity with increasing angle of incidence for the martian Nakhlite analog glass for 30 s measurements and a pressure of 10^3^ Pa, measured every 5° ± 2° increments between 0° and 15°. For clarity and ease of comparison to the distance test, intensity scale (counts per second) and symbology is identical to Figure 13. Generally, the angle of incidence is less a of a concern regarding loss of signal quality; however, some elements (Si, Al) seem to be more affected than others (Fe, Ca, Ti, Mn).

We also assessed the changes in count rates (and therefore data quality) for the portable XRF depending on the angle of incidence (of the beam) to the sample surface ranging from 0 to 15°. The results, again measured on the Nakhlite glass, are summarized in Figure 11, showing that the angle of incidence minimally alters the count rate, especially when compared with the influence of measurement distance shown in Figure 14 on the same (intensity) scale. Interestingly, the matrix elements Si and Al appear to be more affected by the angle of incidence, indicated by the steep drop between 5 and 10°, compared with other elements such as Fe, Ca, or K. The overall intensity at 15° for K, Ti, Mn, Ca, and Fe is roughly 73% ± 5% compared with the instrument being aligned orthogonally (0°) to the sample surface, whereas the intensity for Si and Al is only 24% ± 4% over the same range.

In summary, XRF analyses are sensitive to the way the particular unit is held against the sample surface, especially with respect to the matrix elements Si and Al.

## 4. Discussion

### 4.1. Comparison of in-sim data quality to standardized out-of-sim conditions

The vis-NIR spectrometer performs well during EVAs, as the light source is powerful enough to overcome typical gaps and vesicle sizes (less than 15 mm). Since there are no methodical differences in operating the vis-NIR spectrometer in the field and in a more controlled (laboratory) environment, the data quality is virtually the same, so long as the instrument is not used in direct sunlight more than 15 mm from the sample.

In contrast, the XRF data quality is strongly influenced when in poor contact with the sample surface, caused by uneven surfaces (*e.g.*, high points, vesicles, void space). The distance test demonstrated that analyzing samples with gaps or vesicles greater than 4 mm causes significant deviations from the true composition (outside the RMSD). Therefore, we recommend that field analyses are prepared (creating flat surfaces, removing unwanted residue on the surface such as dust) during the EVA while controlling atmospheric conditions around the detector (weak vacuum). By doing so, this will also mitigate the quality loss. Although spectral improvement can also be achieved via helium purging, this is risky to do because the exposed, −15°C cooled detector is exposed to dust and gases/volatiles (*e.g.*, active fumaroles fumes, water vapor on humid days) that would condense onto the detector and cause contamination and possible irreparable damage to the instrument.

### 4.2. Most and least influential parameter for spectral quality

The single most influential parameter that affects data quality for both vis-NIR and XRF analyses is the distance of the instrument to the rock surface and, therefore, also the size/depth of the vesicles in the samples. We note that the ASD Halo data is less affected than the XRF by instrument distance.

The least influential parameter for spectral quality in this study is the angle of incidence, as long as the instrument is held within 15° to normal of the sample surface.

### 4.3. Mineral identification and visual cues

Within the context of our EVAs and the associated decision-making framework, it is important to reduce uncertainty and increase diagnostic capabilities. The MSC and Earth-based science team has to contend with a limited situational awareness of the EV crew surroundings, while simultaneously managing their paramount requirement for sample collections that will meet their science objectives.

Our field-based results (Fig. 5) suggest that, within the field sampling framework of having to efficiently identify and differentiate unaltered/less altered and highly altered basalt samples, (1) visual color cues (*e.g.*, dark vs. bright surface colors) can be used as an effective means of discerning alteration state, and (2) ASD mineral output can be used as a diagnostic capability to rapidly and broadly categorize basalts into two categories (unaltered/less altered and altered). Further, within these two categories, our results suggest that to fine-tune sample high grading further analyses would be required either in the field using complementary spectral capabilities or in the lab where samples could be prepared for more refined interrogation.

### 4.4. Ease of instrument usage during EVAs

#### 4.4.1. Ergonomics

Handheld instruments were assessed for ease of use *in situ*, during an EVA as opposed to a lab environment. We considered the instruments' design, including weight (which would be reduced on the lunar and martian surface) and size, and whether or not these were disadvantages during EVAs.

Both spectrometers are ∼2.5 kg each (on Earth), which added extra weight to the loads carried during an EVA. The instruments, however, were not always carried by the EV crew that used them, as support crew was present and responsible for carrying some of the instruments. When instruments were in the hands of the EV, one visible limitation was the inability to place instruments down to rest. In the field, resting surfaces are sparse (*e.g.*, avoiding location contamination, inclined surfaces where instruments slide downslope). This means that instruments were sometimes constantly in hand for expedient use between applications. Future studies of human fatigue among EVA crew may consider the periods of activity (human energy use) when instruments are in hand.

The instruments' design in relation to their object(s) of study was a human-technology relationship we considered as a matter of ergonomics. The EV crews' ability to place instruments for use was sometimes obstructed by the instrument's design.

**FIG. 15.**
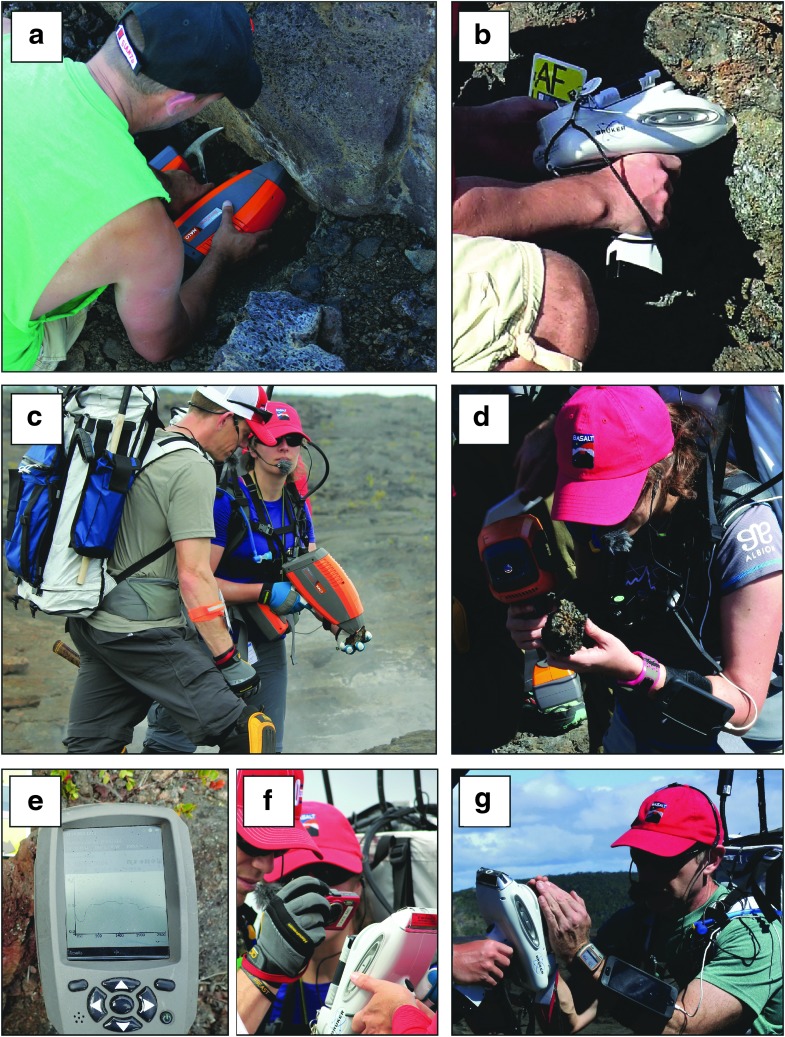
**(a)** Using the vis-NIR out-of-sim at the lower portion of an outcrop, for which a hole had to be dug to be able to measure the basal part of the outcrop; **(b)** positioning the XRF unit near the outside of a hole (located in the shade), in which the instrument did not fit; **(c)** holding rock in hand to avoid measurement too close to steaming fumarole; **(d)** preference to hold rock in hand for easier measurement to avoid kneeling on the ground; **(e)** acquiring photographs of the vis-NIR instrument result display after each to transmit the spectral data to the SST crew due to the inability of the unit to stream the data directly into an online database (note the glare of the screen when taking photos); **(f)** taking photos of XRF result screen in case connectivity issues to stream data directly into online database; **(g)** difficulty in reading XRF display during an EVA in direct sunlight.

For example, we noted that it was hard to reach certain spots at outcrops with the instruments, such as narrow cracks with widths and depths on the centimeter scale. In these cases, the instrument nose, mouth was too large and could not reach the targets of interest. The EV had to hand off the instrument (or find a place to rest it), take time to break up rocks, and recover a sample surface of interest on which to use the instrument (Fig. 15a–d). From a planetary exploration perspective, if the EV crew deploys from a rover (pressurized or unpressurized) (Ross *et al.*, 2013) and does not travel a great distance from the rover (<200 m), then the handheld instruments could remain on a tool deck or locker on the rover and be retrieved when needed. However, in anticipation that crews will walk hundreds of meters away from a rover or habitat and remain on EVA for several hours, final designs for handheld instruments need to be optimized for mass (even in reduced gravitational environments on the Moon, Mars, or micro-gravity bodies).

In addition, instruments will need to be easily operable by people wearing space suit gloves and have user interfaces that are readable in harsh environments. These design requirements will be critical for implementation of handheld instruments into sample science operations. To mitigate some of these problems with respect to instrument size, future iterations of scientific analytical spectrometers could provide easier handling by reducing their size to better fit in smaller or hard-to-reach places/outcrops, fiber optics cable (for the vis-NIR) that can extend into higher or lower lying parts of outcrops, as well as into cracks or void spaces. In addition, size reduction would allow the instruments to be strapped around the astronauts' waist or stored in pockets/compartments within the EVA suit. We observed that the holster for the vis-NIR was effectively never used, and that it was easier to carry the instrument by hand rather than having a blocky object attached to the hip, especially when wearing a backpack.

During EVAs, assessing the effectiveness of an instrument was not limited to its capability to draw a measurement from a basalt rock but also included crews' (all teams) ability to see the instruments' data (*e.g.*, on the instrument's display face), to quickly transmit (receive) data, to manipulate data using software, and to interpret data (Mirmalek *et al.*, 2017).

#### 4.4.2. Connectivity

The BASALT analog mission is supported by an online database called xGDS, to which data were streamed and are archived (Marquez *et al.*, 2019). This database also supports the integration of our instruments, as long as they have untethered streaming capability (*i.e.*, built-in Wi-Fi or Bluetooth) or ports to which such capabilities can be attached (*e.g.*, insert a Wi-Fi-connected memory card).

The vis-NIR instrument is unable to live-stream data into the xGDS database. After 1 to 2 min per scanning a single candidate sample location (including time used to decide for multiple different measurement spots), the only way to transfer data from the instrument is to power it down, attach it via cable to a computer, power it on again, and synchronize the data to the computer with a specialized data-managing software. This transmitting process requires several minutes, therefore consuming precious time that was not well budgeted for in an EVA.

We developed a simple workaround: The data output displayed on the internal display was first orally read by the EV crew, and then photos of the screen (mineral identification and entire spectrum) were taken and transferred to the online database (Fig. 15e). This workaround is not perfect because a photo of the small screen is not as good as data, which could be quantitatively inspected and manipulated by the IV and SST crew. In addition, it requires extra time to acquire usable photographs of the ASD Halo's screen in bright sunlight or where strong reflections are present. Once acquired, the photograph can be several MB in size, requiring several tens-of-seconds or minutes to transfer during limited-bandwidth EVA missions.

The XRF, in contrast, provides the capability to insert a Wi-Fi memory card to which data can be automatically saved and live-streamed to the database. The data files were small (∼10 KB) and rapidly uploaded. Although it was not always necessary, the workaround of reading the instrument data aloud was used for the XRF, until the IV team recognized and requested that EV cease providing the extra description.

We find that integrating scientific instruments into online databases (*i.e.*, data streaming) greatly enhances science investigations in analog exploration missions. It reduces the duration time between data acquisition in the field and data arrival in the MSC. Future instruments designed specifically for planetary exploration will certainly be optimized to include the ability to stream the data directly to the local network. Data clarity increases with a decrease in communication interruptions and problems with low image readability due to reflections and sunlight. With respect to data transfer, we recommend that handheld planetary instruments be able to not only stream data wirelessly but also save data internally for redundancy and backup and preserve any files that are too large to stream across the network. It should be noted that the oral description can be a reliable but time-consuming workaround, if audio communication is an option.

### 4.5. Comparison to results of other studies using portable field spectrometers

#### 4.5.1. Olympus Innov-X DELTA premium

Young *et al.* (2016) evaluated a handheld XRF for its usefulness in the field. The evaluated XRF model was an Olympus Innov-X DELTA Premium. The X-ray tube (4W Rhodium) and silicon detector were comparable to our instrument, and the same array of major element oxides were investigated (MgO through Fe_2_O_3_). Some of our results from the lab and the simulated EVAs are consistent with their general findings, including measurement time and distance effect collected during both lab and geological field work.

Similar to our instrument, their handheld XRF analyzer provided accurate data (within 3% uncertainty) for measurement times of 60 s and longer. These results are for flat/sawed rock surfaces spanning a composition from andesite to Si-undersaturated basalt, which are unaltered and largely homogenous (containing only a few larger phenocrysts and vesicles).

Young *et al.* (2016) also investigated the influence of surface roughness, which equates to increasing instrument-sample distance. Without a detailed description of how their surface roughness correlates to an absolute (sample-instrument) distance, as provided in this study, their results show that increasing roughness at identical conditions (time, beam intensity etc.) reproduces the results of the flat surfaces within 2%, although errors on rough surfaces appear to be almost in all cases twice as large compared with flat surfaces, presumably due to scattering.

#### 4.5.2. vis-NIR HALO

We assessed how the reflectance spectra of the portable (field) unit compare with a laboratory vis-NIR instrument. Lab measurements were collected in a dark room under ambient conditions by using an ASD FieldSpec 3 spectroradiometer at Arizona State University, Tempe.

Samples were illuminated with a quartz tungsten halogen source, and reflected light was collected through a fiber optic cable supplied with the spectroradiometer. Both the fiber optic cable and the light source were mounted on a goniometer to enable collection of data with an observation geometry identical to the Halo TerraSpec (*i* = 0 and *e* = 23). Data were collected, processed to reflectance relative to Spectralon, and corrected for Spectralon absorption irregularities as well as detector offsets. Samples used for this assessment were powdered and sieved (particle sizes <75 microns) basalts collected at Craters of the Moon, Idaho.

**FIG. 16.**
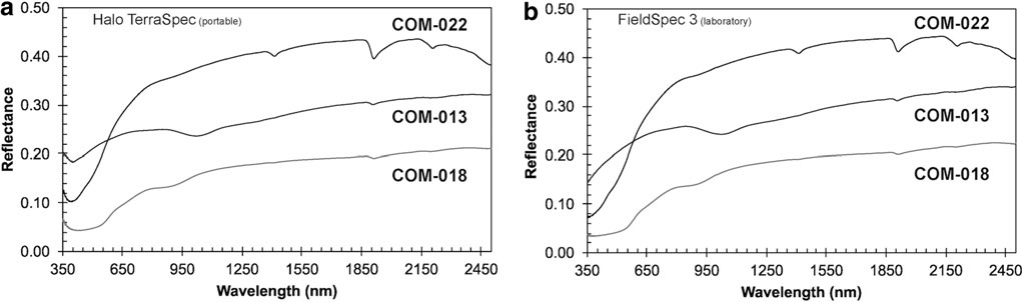
**(a)** Field unit to **(b)** laboratory unit comparison. Shown are data of three samples collected at Craters of the Moon, measured with the portable ASD (Halo TerraSpec) and laboratory setup (FieldSpec 3). COTM013 is composed of olivine and glass (likely devitrified/hydrated); COTM018 contains hematite, glass, and minor clay; and COTM022 contains goethite/ferrihydrite and clay (Fe-bearing smectite).

Data for both instruments are plotted in Figure 16. We find that the Halo TerraSpec (portable unit) provides almost identical spectra to the laboratory setup (FieldSpec 3). Only differences are observed in the UV-blue part of the spectra, perhaps due to different lighting conditions (Halo TerraSpec were not measured in the dark room). However, this spectral range is mostly irrelevant for our analysis and interpretation to infer mineralogy.

Our findings are broadly consistent with the study of Bishop *et al.* (2016). They used a Panalytical TerraSpec HALO vis-NIR mineral identifier to study the field spectra collected on outcrops of altered volcanic material from Kīlauea volcano (Big Island, Hawai'i) and Polihua Trail (Lanai, Hawai'i), and they compared these field spectra with spectra collected in the laboratory with an ASD FieldSpec Pro.

The alteration mineralogy in these outcrops includes opal, jarosite, and gypsum. Bishop *et al.* (2016) found that laboratory spectra show stronger signatures of opal and jarosite compared with measurements done in the field, which seem to overcast the Fe^3+^ absorption feature. The study also systematically investigated the influence of grain size (ranging from 150 μm up to 1 mm) in the laboratory and compared the results with the spectra collected in the field. They show that with increasing grain size the reflectance decreases whereas absorption features remain preserved. In summary, spectra acquired in the field are broadly consistent (*e.g.*, presence of absorption features) with spectra acquired in the lab.

### 4.6. Identification of alteration states in basaltic terrains using handheld spectrometers

**FIG. 17.**
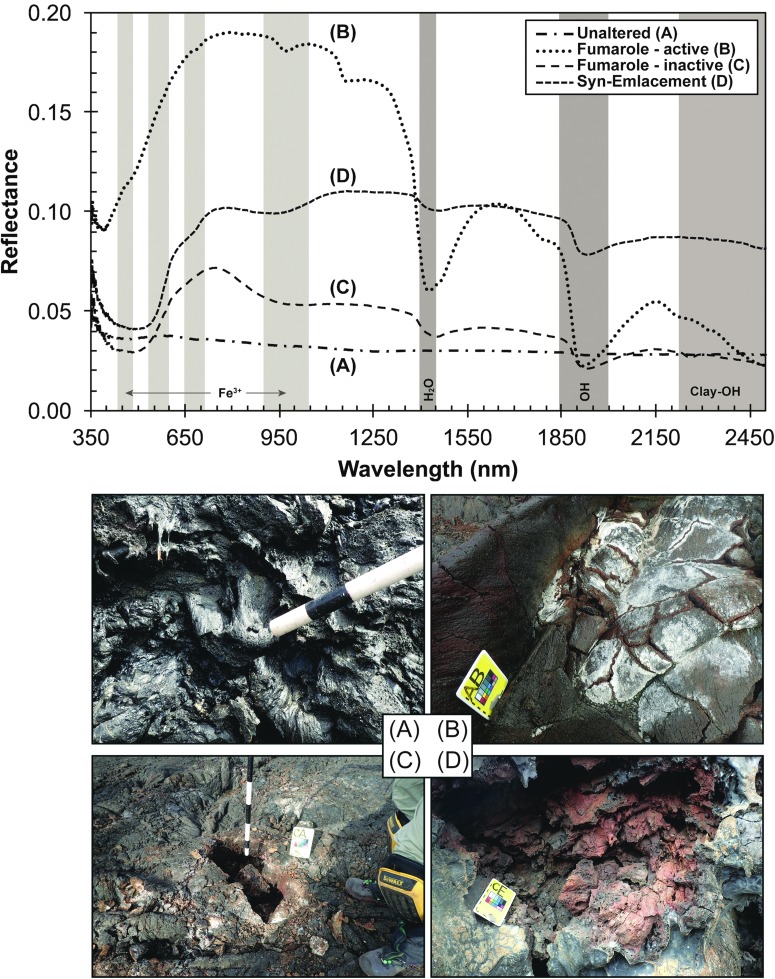
vis-NIR field spectra of alteration states and their major absorption bands (gray bands: Fe^3+^, OH, H_2_O, Clay-OH), as well as photographs of representative outcrops (bottom): **(A)** Unaltered basalt with black and glassy groundmass, **(B)** active and **(C)** inactive Fumarole with zeolites, clays, and hydroxides, **(D)** Syn-Emplacement alteration with bright red color due to oxidation. For detailed descriptions and classifications of encountered alteration states, see Section 4.6 and Hughes *et al.* (2019).

Examples of encountered alteration states, including collected vis-NIR spectra and XRF data, are given in Figure 17 and Table 4, respectively.

**Table 4.** Field Analysis of Samples of Selected Alteration States in wt% Provided by the pXRF

	*SiO_2_*	*TiO_2_*	*Al_2_O_3_*	*FeO*	*MnO*	*MgO*	*CaO*	*Na_2_O*	*K_2_O*	*P_2_O_5_*	*Total*
A (unaltered)	50.81	1.74	10.55	14.37	0.22	10.92	8.89	1.58	0.66	1.15	100.89
B (fumarole-active)	57.62	n.d.	7.56	16.93	0.29	6.66	5.98	2.63	0.76	0.71	99.15
C (fumarole-relict)	56.10	n.d.	6.31	13.34	0.23	4.97	8.30	3.30	0.68	n.d.	93.23
D (Syn-emplacement)	53.87	1.44	9.42	14.75	0.23	9.23	7.43	2.22	1.08	0.96	100.64

*Unaltered basalt:* Our field and laboratory tests show that unaltered basalt (Fig. 17A) is easily identified by a low-reflectance vis-NIR spectrum that typically lacked any absorption features. If bulk chemistry of unaltered basalt is already known (*e.g.*, deployed rover analyses before the arrival of crewed missions), then the XRF analyses can provide independent assessments in addition to the vis-NIR measurement. In any case, we recommend analyzing several spots of black basalt in the proposed target regions, which provides a baseline from which type of alteration can be assessed:

*Fumaroles*, active versus relict*:* Temperature measurements, such as using a forward-looking IR (FLIR) camera, provided a quick and easy assessment to detect heat signatures above the ambient (ground) temperature (active fumarole). Both active and relict fumaroles are associated with a wide variety of mineral deposits, indicated by OH, H_2_O absorption bands in vis-NIR spectra due to the presence of zeolites, clays, and hydroxides. In our field tests, active fumaroles (Fig. 17B) appear much brighter (high-reflectance spectra) compared with unaltered basalt, and more reflective than inactive fumaroles (Fig. 17C). The brightness difference between active and relict fumarole may be a consequence of weathering processes on the surface over time, which dilutes and removes the mineral deposits at active fumaroles once the fumarole ceased outgassing.

On Earth, active volcanism exhales sulfuric gasses, leading to bright yellow-greenish (elemental) sulfur deposits. Even though the XRF unit was not calibrated to quantify sulfur in our use cases, the SST used the raw spectra to check the presence of a sharp Kα spectral line at ∼2.309 keV, indicating the presence of sulfur (either elemental or in mineral form, such as gypsum). The raw XRF spectrum is highly diagnostic to discern whether the yellow coloration resulted from sulfur deposits or mineralization (mixes) with similar coloration (such as clays and hydroxides).

Syn-emplacement alteration (Fig 17D) was identified by visual cues, mostly a bright red color resulting from oxidation at high temperatures during lava emplacement. We used the vis-NIR spectrometer to check for Fe^3+^absorption bands located between ∼400 to 1100 nm, indicating the presence of hematite (Fe_2_O_3_). We expected to see strongly elevated FeO values (relative to the unaltered basalt); however, this was not always the case (see Fig. 17D and XRF analysis in Table 4).

Finally, during a single EVA, several locations were identified as the same type of alteration (active fumarolic deposits etc.), whereby the sample high grading in these location was driven by two main aspects: (1) the ease of sampling, especially with regard to assure sterility of the sample location and recovered samples for biological and biochemical studies, and (2) the quality of characterization by the handheld instruments, such as band depths, variety of absorption features, elevated/depleted elemental abundances relative to unaltered basalt, peak presence, and height in XRF spectra.

In conclusion, spectrometers provided rapid *in situ* assessment of the mineralogy, as well as elemental abundances and elemental signatures of basaltic rocks and their alteration states. Therefore, they are useful for high-grading samples in the field.

### 4.7. The future of human exploration missions with handheld spectrometers

Our assessment of these two spectrometers shows the strengths and weaknesses of these scientific instruments from multiple perspectives: technical and scientific capabilities, design, and ergonomics.

Though the instruments proved to be very useful in the field, we experienced limitations in using the current generation of handheld spectrometers. We identify a few ways to approach these limitations: employing a very detailed instrumentation protocol that would support EV crew in recognizing and communicating intrinsic sample variables to apply specific mineral calibrations and interpretations (such as shown in Supplementary Fig. S2); development of instrument design (*e.g.*, increased insensitivity to sample distance, better sensitivity to lighter elements by applying filters and/or larger detectors); and adding more equipment (*e.g.*, rock preparation tools, spectrometers) to the instrument suite.

In particular, iterations of future handheld spectrometers that combine two or more techniques into one may provide some advances: (1) reduction of the number of instruments carried around (by either the EV crew or field support team), (2) reduction of measurement time so that multiple instruments (*e.g.*, vis-NIR, XRF, LIBS, Raman) do not need to be precisely repositioned to match the identical analysis spot for each measurement. The saved time may be used to characterize more locations in larger areas instead.

## 5. Conclusions

Handheld scientific instrumentation provides *in situ* analyses during exploratory field campaigns, characterizing geological samples in terms of their mineralogy (by vis-NIR spectroscopy) and quantitative and qualitative geochemical analyses (by XRF spectroscopy) in real time. Here, we would like to restate and answer our set of geological and human-technology research questions on instrument evaluation and implementation of capabilities in a (simulated) EVA architecture aiding the development of protocols for sample high grading and triage during future crewed missions to the lunar and/or martian surface.

(1) *Could differences in degree of alteration be effectively assessed through visual cues?* Various colorations on rock surfaces are indicative of a variety of mineral depositions, ranging from unaltered (black) basalt to vibrant colors (red and yellow) at highly altered sites, such as active fumaroles and alteration that occurred during lava emplacement.
(2) *How would each instrument best support visual cues that identify alteration*? Both instruments provided information in support of visual cues to identify different types of alteration. Location of absorption bands was most helpful, whereby the lack of absorption bands paired with low reflectivity indicated unaltered, fresh basalt. Auto-mineral identification software provided only broad assessment relating to the presence of mineral groups (*e.g.*, clays, hydroxides) rather than accurately distinguishing between specific minerals within the same mineral group. As such, we recommend doing spectra analysis manually.

Elemental analyses provided by the XRF were useful to validate interpretations based in vis-NIR data rather than confirmation of visual cues. Attention was paid to the shift to higher/lower elemental abundances (in comparison to unaltered basalt) for key elements that correspond to specific minerals (*e.g.*, elevated FeO for Hematite; elevated SiO_2_ and Al_2_O_3_ with depleted MgO, indicating phyllosilicates). However, the elemental abundance depends on the amount and thickness of the mineral deposit, which is also a mixture with other minerals, which causes the shift in elemental abundances to be not as obvious as one would hope. Therefore, using these two instruments in concert with visual cues provides more robust interpretations than with one instrument alone. Moreover, the sole presence of specific key elements (indicated by peaks at certain keV in the raw spectra), such as sulfur or heavy metals associated for fumarolic deposits, was found to be very diagnostic.

**FIG. 18.**
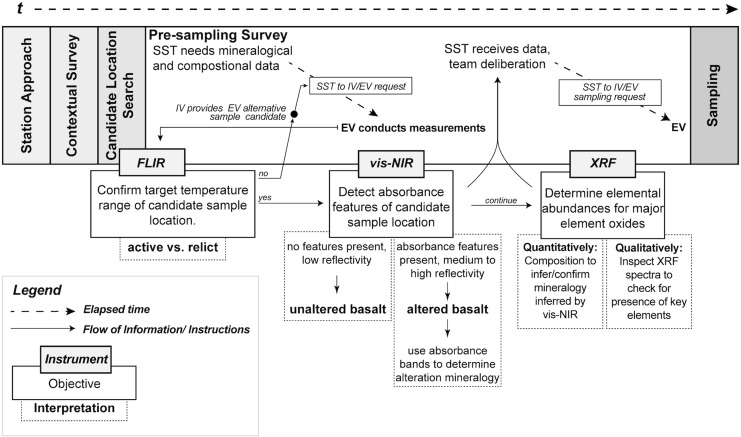
Representation of integrating instruments within EVA architecture, during the presampling survey phase. Within the presampling survey phase, the sequence of events begins with the objective that the “SST needs mineralogical and compositional data,” which are sent to the IV team that communicates them to the EV team. In the next event, “EV conducts measurements,” a selection of instruments is used in a specific order, which for BASALT objectives were an FLIR spectrometer to measure temperature, vis-NIR, and XRF. If the FLIR confirms the objective (yes), then EV moves on to use the vis-NIR; if the FLIR does not confirm the objective, then EV receives instruction to move to next candidate location by IV (as per SST recommendations). While the EV team is using the vis-NIR and then the XRF, the SST receives data over which they deliberate to produce a sample priority list for the next phase (phase 5, sampling). Time is represented with a singular arrow; however, for Mars missions and analog missions, multiple timelines run simultaneously as some teams (SST) are on Earth time and some teams (EV/IV) are on Mars time, as well as there is the consideration of communication latency among them. Note that FLIR measurements are also used during phase 3 (Sample Location Search). EV, extra-vehicular.

With respect to human-technology relationships:

(1) *How does a handheld scientific instrument serve the EVA sample prioritization process both in the field and in the science support team?* The workflow in Figure 18 offers a representation of instrument suite deployment during an EVA. It includes, with contextual details of team communication and activity times, and it conveys the relationship between instruments, technology, and the science process in an EVA. The intent of the representation is to highlight key aspects of human and technology interactions within the activity of integrating instruments into EVA architecture, presampling survey phase, with attention to mission timelines, information flow, and scientific analysis.
(2) *What are the most critical conditions—analytical measurement and data transmission—influencing data quality?* We find that the sample condition itself, such as uneven surfaces preventing the instruments from flat on contact with the sample surface, is critical. As demonstrated, analyzing surfaces with gaps/voids of greater than 4 mm results in radical loss of quality to quantifying elemental abundances with the tested XRF unit. Poor data quality, if unnoticed, incurs the chance of false interpretations, risking the success of returning the appropriate samples that are specific to the EVA objective. This issue feeds directly into our follow-up question.
(3) *Given the scientific objectives, what type and how many instruments are optimal during EVAs?* We advocate for the inclusion of at least one other (independent) analysis method that provides more robust data under the same measurement conditions, such as the vis-NIR, to interpret rock with respect to the science objective. During all of our EVAs, we never relied on only one technique (*e.g.*, only XRF measurements).
For example, we encountered outcrops that were steaming, but questions remained as to whether this was due to magmatic outgassing or recycled meteoric water. The mineralogy based on vis-NIR alone was not indicative enough to make satisfactory decisions; however, qualitative assessment by the XRF (detecting certain key elements hinting toward magmatic origin, such as sulfur) helped to come to a satisfactory conclusion in such dubious cases. Further studies are required to investigate the usefulness of three or even more scientific instruments during EVAs, such as Laser-Induced Breakdown (LIB) and Raman spectrometers, which are part of the payload of current and/or future Mars Exploration Rover missions (*e.g.*, Curiosity, Mars 2020, ExoMars).
(4) *How can instrument use be synchronized to leverage relative strengths of multiple tools?* Our answer to this question resonates with the aforementioned aspects to obtain satisfactory data quality to make robust interpretations. Incorporating these instrumentations in ∼25 individual (simulated) EVAs during field deployments in 2016 and 2017, we found that the use of two instruments in a particular sequence worked well. The established sequence of instrument use began with obtaining vis-NIR spectra, as the method itself was fairly robust (*e.g.*, results shown for the distance and angle test) for measurements on rocks of basaltic nature (unaltered and altered states). Interpretations based on these data were then cross-checked with quantitative and qualitative geochemical data from the XRF (Fig. 18).

In conclusion, both the vis-NIR and XRF field spectrometers were valuable additions to extravehicular activities, as they provide rapid, *in situ* measurements. Due to their construction and method, some of these analysis techniques are currently more robust than others. Considering the results of the tests conducted in this study, the data quality can be increased and maintained on a level at which these instruments return scientific value for sample prioritization during an EVA.

## Supplementary Material

Supplemental data

Supplemental data

Supplemental data

Supplemental data

## Abbreviations Used

ASD: Analytical Spectral Devices
BASALT: Biologic Analog Science Associated with Lava Terrains
ConOps: Concepts of Operations
COTS: commercial off-the-shelf
EV: extravehicular
EVAs: extravehicular activities
FLIR: forward-looking IR
IV: intravehicular
LIB: laser-induced breakdown
MIR: mid-infrared
MSC: Mission Support Center
RMSD: root-mean-square standard deviation
SST: Science Support Team
UV: ultraviolet
vis-NIR: visible-near infrared
XRF: X-Ray Fluorescence
