# Supplementary material for: Requirements for Portable Instrument Suites during Human Scientific Exploration of Mars
Source: Astrobiology. 2019 Mar 6;19(3):401–25. doi: 10.1089/ast.2018.1841 (PMC6442242; doi:10.1089/ast.2018.1841)
Supplement: Supplemental data [file Supp_Table1.zip › SupplTable1.docx]

| DATE: | 9-Aug-16 | START TIME: | 14:05 MT | END TIME: | 19:18 MT |  |
| --- | --- | --- | --- | --- | --- | --- |
| **GRID** | **COLOR** | **VESSICLES (cm or mm scale; %age)** | **OTHER** | **SCAN 1** | **SCAN 2** | **SCAN 3** |
| A1 | Red, black, organge; but dark compared to others | none on surface, outer skin |  | Clinop, 2 stars | Clinop | Clinop |
|  |  |  |  | VISNIR, no match | Mg Illite | Magnesite |
|  |  |  |  |  |  |  |
| B1 | Orange, black, maroon | stretched vessicles on outer surface, few mm | Flat spectra; hard to get onto surface; | No match found | No match found | No match found |
|  |  |  |  |  |  |  |
|  |  |  |  |  |  |  |
| C1 | Mostly black | vessicles are 1-3mm; 40% | lichen around it, but not shooting on the lichen | No match found | No match found | No Match found |
|  |  |  |  |  |  |  |
|  |  |  |  |  |  |  |
| D1 | Dark red, black, blue sheen | no vessicles discernible |  | No match found | No Match found | No match found |
|  |  |  |  |  |  |  |
|  |  |  |  |  |  |  |
| E1 | grass: skip |  |  |  |  |  |
|  |  |  |  |  |  |  |
|  |  |  |  |  |  |  |
| F1 | black blue, with small spot of dark red, lihen around it | no vessicles discernible |  | No match found | No match found | No match found |
|  |  |  |  |  |  |  |
|  |  |  |  |  |  |  |
| G1 | black blue, with small spot of dark red, some white deposit on it | stretched vessicles on outer surface; few mm |  | No match found | No match found | No match found |
|  |  |  |  |  |  |  |
|  |  |  |  |  |  |  |
| H1 | black blue, with small spot of dark red (that looks like lichen), some white deposit on it | stretched vessicles on outer surface; few mm | this is up the outcrop face from G1 | No match found | No match found | No match found |
|  |  |  |  |  |  |  |
|  |  |  |  |  |  |  |
| I1 | Dark, black, blue; red stains in vessicle | mm scale vessicles, red stains inside; 50% coverage | this is up the outcrop face from G1 | No Match found | No match found | No match found |
|  |  |  |  |  |  |  |
|  |  |  |  |  |  |  |
|  |  |  |  |  |  |  |
| **GRID** | **COLOR** | **VESSICLES (cm or mm scale; %age)** | **OTHER** | **SCAN 1** | **SCAN 2** | **SCAN 3** |
| A2 | Orange, tan, very red | none discernible | Very akward overhang at bottom of lobe | Fe smectite | Clinop | Clinop |
|  |  |  |  |  | Mg Ilite | No Match |
|  |  |  |  |  | Tourmaline, Magnesite |  |
| B2 | Dark brown, maroon | stretched out vessicles | the surface is flat | No Match found | No Match Found | No match found |
|  |  |  |  |  |  |  |
|  |  |  |  |  |  |  |
| C2 | Blueish black, dark, maroon | stretched out vessicles |  | No match found | No match found | No match found |
|  |  |  |  |  |  |  |
|  |  |  |  |  |  |  |
| D2 | Maroon colored, brighter than C2 | mm-scale vessicles; not a lot of them | cm gap between rock and ASD, because the rock is concave | No match found | No match found | No match found |
|  |  |  |  |  |  |  |
|  |  |  |  |  |  |  |
| E2 | bush:  skip |  |  |  |  |  |
|  |  |  |  |  |  |  |
|  |  |  |  |  |  |  |
| F2 | Dark red, with dark blue |  |  | No match found | No match found | No match found |
|  |  |  |  |  |  |  |
|  |  |  |  |  |  |  |
| G2 | Mostly black and blue, with some spots of red with some orange lichen |  |  | No match found | No match found | No match found |
|  |  |  |  |  |  |  |
|  |  |  |  |  |  |  |
| H2 | Black, Blueish red with some white, maroon | stretched vessicles that almost look like cracks |  | No match found | No match found | No match found |
|  |  |  |  |  |  |  |
|  |  |  |  |  |  |  |
|  |  |  |  |  |  |  |
| **GRID** | **COLOR** | **VESSICLES (cm or mm scale; %age)** | **OTHER** | **SCAN 1** | **SCAN 2** | **SCAN 3** |
| A3 | orange tan, | mm-scale 50% |  | clinop | clinop | clinop |
|  |  |  |  | tourmaline | tourmaline | tourmaline, 1 star |
|  |  |  |  |  | dolomite, 1 star | magnesite, 1 star |
| B3 | dark brown with red mixed in | stretched out vesicles |  | No match found | No match found | No match found |
|  |  |  |  |  |  |  |
|  |  |  |  |  |  |  |
| C3 | black | flat, glassy surface, mm-vesicles 80% |  | No match found | No match found | No match found |
|  |  |  |  |  |  |  |
|  |  |  |  |  |  |  |
| D3 | black | flat, glassy surface, mm-vesicles 80% |  | No match found | No match found | No match found |
|  |  |  |  |  |  |  |
|  |  |  |  |  |  |  |
| E3 | dark, black | stretched out mm-cm-scale vesicles, lichen |  | No match found | No match found | No match found |
|  |  |  |  |  |  |  |
|  |  |  |  |  |  |  |
| F3 | dark black, glassy | stretched vesicles, flat, no lichen, |  | No match found | No match found | No match found |
|  |  |  |  |  |  |  |
|  |  |  |  |  |  |  |
|  |  |  |  |  |  |  |
| **GRID** | **COLOR** | **VESSICLES (cm or mm scale; %age)** | **OTHER** | **SCAN 1** | **SCAN 2** | **SCAN 3** |
| A4 | not possible |  |  |  |  |  |
|  |  |  |  |  |  |  |
|  |  |  |  |  |  |  |
| B4 | maroon, with black throughout | highky vesiculated 90%, mm-cm-scale vesicles | Lobe color seems to change from dark ornage to strong red | Hematite*** | Hematite*** | Hematite*** |
|  |  |  |  | Fe-Mont** | Fe-Mont** | Fe-Mont** |
|  |  |  |  |  |  |  |
| C4 | Browny, reddish marron | highly vesiculated 90%, mm-cm scale vesicles |  | No match found | No match found | No match found |
|  |  |  |  |  |  |  |
|  |  |  |  |  |  |  |
| D4 | Browny, reddish marron | highly vesiculated 90%, mm-cm scale vesicles | same surface as before | No match found | No match found | No match found |
|  |  |  |  |  |  |  |
|  |  |  |  |  |  |  |
| E4 | Dark black with lichen | vesicles, very vesicular |  | No match found | No match found | No match found |
|  |  |  |  |  |  |  |
|  |  |  |  |  |  |  |
| F4 | darl black glassy surface | stretched vesicles | some lichen, trying to shoot outside | No match found | No match found | No match found |
|  |  |  |  |  |  | Spectrum_0078 |
|  |  |  |  |  |  |  |
|  |  |  |  |  |  |  |
| **GRID** | **COLOR** | **VESSICLES (cm or mm scale; %age)** | **OTHER** | **SCAN 1** | **SCAN 2** | **SCAN 3** |
| A5 | not possible |  |  |  |  |  |
|  |  |  |  |  |  |  |
|  |  |  |  |  |  |  |
| B5 | Bright red, with some gray black | mm-cm-scale vesicles, 90% |  | Clinop*** | Clinop*** | Clinop*** |
|  |  |  |  |  |  |  |
|  |  |  |  |  |  |  |
| C5 | dark black very glassy | cm-scale vesicles, sprakly bits in vesicles, 90% and stretched out |  | No match found | No match found | No match found |
|  |  |  |  |  |  |  |
|  |  |  |  |  |  |  |
| D5 | dark black very glassy | cm-scale vesicles, sprakly bits in vesicles, 90% and stretched out | same as before | No match found | No match found | No match found |
|  |  |  |  |  |  |  |
|  |  |  |  |  |  |  |
| E5 | Dark brown weathered | stretched out vesicles | rock face that's very weathered, no lichen were measured detecable with naked eye but lichen around meaasurement spot | Clinop*** | Harmatome** | No match found |
|  |  |  |  | Magnesite** | Magnesite* |  |
|  |  |  |  | Mg-Illite* |  |  |
| F5 | dark glassy | stretched out vesicles |  | No match found | No match found | No match found |
|  |  |  |  |  |  |  |
|  |  |  |  |  |  |  |
|  |  |  |  |  |  |  |
| **GRID** | **COLOR** | **VESSICLES (cm or mm scale; %age)** | **OTHER** | **SCAN 1** | **SCAN 2** | **SCAN 3** |
| A6 | Maroon | very small vesicles |  | No match found | No match found | No match found |
|  |  |  |  |  |  |  |
|  |  |  |  |  |  |  |
| B6 | marron with some black | cm-scale vesicles, ~90% |  | No match found | No match found | No match found |
|  |  |  |  |  |  |  |
|  |  |  |  |  |  |  |
| C6 | little more black, brownish red, glassy looking | cm-scla vesicles, |  | No match found | No match found | No match found |
|  |  |  |  |  |  |  |
|  |  |  |  |  |  |  |
| D6 | same as before | same as before |  | No match found | No match found | No match found |
|  |  |  |  |  |  |  |
|  |  |  |  |  |  |  |
| E6 | glassy stretched out surface, black and rust colored, underneath its somewhat tan colored | stretched surface, snake skin | stretched upper (horizontal) surface. Took picture (time 15:54) | Clinop*** | Clinop*** | Clinop*** |
|  |  |  |  |  |  |  |
|  |  |  |  |  |  |  |
|  |  |  |  |  |  |  |
| **GRID** | **COLOR** | **VESSICLES (cm or mm scale; %age)** | **OTHER** | **SCAN 1** | **SCAN 2** | **SCAN 3** |
| A7 | rust red color | cm-sclare vesicles everywhere |  | No match found | No match found | No match found |
|  |  |  |  |  |  |  |
|  |  |  |  |  |  |  |
| B7 | red rust color again | flat surface, no discernable vesicles | ~0.5cm gap between rock surface and gun window | Clinop | Clinop** | Clinop** |
|  |  |  |  |  | Dolomite* | Dolomite* |
|  |  |  |  |  | Ankerite* |  |
| C7 | Maroon and black | cm-scale vesicles |  | No match found | No match found | No match found |
|  |  |  |  |  |  |  |
|  |  |  |  |  |  |  |
| D7 | tan reddish, very weathered | lots of vesicles but weathered away |  | No match found | No match found | No match found |
|  |  |  |  | shaking, slipped | S_0119 | S_0120 |
|  |  |  |  | S_0118 |  | S_0121 |
| E7 | red black | highky weathered vesiculated surface |  | No match found | No match found | Goethite** |
|  |  |  |  |  |  |  |
|  |  |  |  |  |  |  |
| F7 | black, glassy | stretched vesicles | surface | No match found | No match found | No match found |
|  |  |  |  |  |  |  |
|  |  |  |  |  |  |  |
|  |  |  |  |  |  |  |
| **GRID** | **COLOR** | **VESSICLES (cm or mm scale; %age)** | **OTHER** | **SCAN 1** | **SCAN 2** | **SCAN 3** |
| A8 | not possible |  |  |  |  |  |
|  |  |  |  |  |  |  |
|  |  |  |  |  |  |  |
| B8 | brightly red | mm-scale vesicles, highly vesiculated, very flat surface |  | No match found | Fe-Mont** | Clinop** |
|  |  |  |  |  |  |  |
|  |  |  |  |  |  |  |
| C8 | Black, glassy | cm-scale vesicles |  | No match found | No match found | No match found |
|  |  |  |  |  |  | S_0133 |
|  |  |  |  |  |  |  |
| D8 | lichen:  skip |  |  |  |  |  |
|  |  |  |  |  |  |  |
|  |  |  |  |  |  |  |
| E8 | lichen:  skip |  |  | Goethite** | No match found | No match found |
|  |  |  |  |  |  |  |
|  |  |  |  |  |  |  |
| F8 | shiny black | stretched out vesicles |  |  |  |  |
|  |  |  |  |  |  |  |
|  |  |  |  |  |  |  |
|  |  |  |  |  |  |  |
| **GRID** | **COLOR** | **VESSICLES (cm or mm scale; %age)** | **OTHER** | **SCAN 1** | **SCAN 2** | **SCAN 3** |
| A9 | red with black coatngs (lichen???), inside is red | lots of vesicles |  | Clinop | Analcime*** | Analcime*** |
|  |  |  |  |  | NH3_IS*** | NH3_IS*** |
|  |  |  |  |  |  |  |
| B9 | reddish, some marron | highly vesiculated, mm-cm-scale |  | Philipsite-Ca*** | Harmotome*** | Philipsite-Ca*** |
|  |  |  |  |  |  | Buddingtonite |
|  |  |  |  |  |  |  |
| C9 | very dark (black) | smooth stretched out surface, no vesicles discernable |  | No match found | No match found | Goethite*** |
|  |  |  |  |  |  | Hematite* |
|  |  |  |  |  |  | _S0145 |
| D9 | lichen:  skip |  |  |  |  |  |
|  |  |  |  |  |  |  |
|  |  |  |  |  |  |  |
| E9 | black | stretched out vesicles | surface (horizontal) | No match found | No match found | No match found |
|  |  |  |  |  |  |  |
|  |  |  |  |  |  |  |
|  |  |  |  |  |  |  |
| **GRID** | **COLOR** | **VESSICLES (cm or mm scale; %age)** | **OTHER** | **SCAN 1** | **SCAN 2** | **SCAN 3** |
| A10 | n/a |  | skipped |  |  |  |
|  |  |  |  |  |  |  |
|  |  |  |  |  |  |  |
| B10 | red with black | highly vesiculated 90%, cm-scale |  | No match found | No match found | No match found |
|  |  |  |  |  |  |  |
|  |  |  |  |  |  |  |
| C10 | lichen: skip |  |  |  |  |  |
|  |  |  |  |  |  |  |
|  |  |  |  |  |  |  |
| D10 | lichen: skip |  |  |  |  |  |
|  |  |  |  |  |  |  |
|  |  |  |  |  |  |  |
| E10 | lichen: skip |  |  |  |  |  |
|  |  |  |  |  |  |  |
|  |  |  |  |  |  |  |
| F10 | black surface | stretched out vesicles | horizaontal surface | No match found | No match found | Jarosite** |
|  |  |  |  |  |  |  |
|  |  |  |  |  |  |  |
|  |  |  |  |  |  |  |
| **GRID** | **COLOR** | **VESSICLES (cm or mm scale; %age)** | **OTHER** | **SCAN 1** | **SCAN 2** | **SCAN 3** |
| A11 | Dark and bright red, little bit black; vessicles are cm-scale and 80-90% coverage | cm-scale, 80-90% |  | Fe-Mont, 2 stars | Analcime, 3 stars | Analcime, 3 stars |
|  |  |  |  | Palygorskite, 1 star | Palygorskite, 2 stars | Palygorskite, 2 stars |
|  |  |  |  |  | Nh3_I/S, 1 star | Buddingtonite, 1 star |
| B11 | Dark and bright red, little bit black; vessicles are cm-scale and 80-90% coverage | cm-scale, 70% |  | No match found | No match found | No match found |
|  |  |  |  |  |  |  |
|  |  |  |  |  |  |  |
| C11 | Dark and bright red, little bit black;patchy black | cm-scale, 70% |  | Clinop, 2 stars | Analcime, 3 stars | Analcime, 3 stars |
|  |  |  |  |  | Nh3_I/S, 2 stars | Nh3_I/S, 2 stars |
|  |  |  |  |  |  |  |
| D11 | lichen: skip |  |  |  |  |  |
|  |  |  |  |  |  |  |
|  |  |  |  |  |  |  |
| E11 | lichen: skip |  |  |  |  |  |
|  |  |  |  |  |  |  |
|  |  |  |  |  |  |  |
| F11 | Black, glassy, faint dark brown patches | stretch skin |  | No match found | No match found | No match found |
|  |  |  |  |  |  |  |
|  |  |  |  |  |  |  |
|  |  |  |  |  |  |  |
| **GRID** | **COLOR** | **VESSICLES (cm or mm scale; %age)** | **OTHER** | **SCAN 1** | **SCAN 2** | **SCAN 3** |
| A12 | Dark and bright red, little bit black; | cm-scale, 90% |  | No match found | No match found | Analcime, 2 stars |
|  |  |  |  |  |  | Vermiculite |
|  |  |  |  |  |  | Ankorite, 1 star |
| B12 | Dark and bright red, little bit black; | cm-scale, 90% |  | Analcime, 3 stars | Philipsite-Ca, 3 stars | Analcime, 3 stars |
|  |  |  |  | Palygorskite, 2 stars |  | Vermiculite, 2 stars |
|  |  |  |  |  |  |  |
| C12 | Dark maroon, almost black | mm- to cm-scale vessicles, bright red inside, 70% |  | No match found | No match found | No match found |
|  |  |  |  |  |  |  |
|  |  |  |  |  |  |  |
| D12 | lichen: skip |  |  |  |  |  |
|  |  |  |  |  |  |  |
|  |  |  |  |  |  |  |
| E12 | lichen: skip |  |  |  |  |  |
|  |  |  |  |  |  |  |
|  |  |  |  |  |  |  |
| F12 | black surface | stretched surface, flat |  | No match found | Jarosite, 2 stars | No Match found |
|  |  |  |  |  |  |  |
|  |  |  |  |  |  |  |
|  |  |  |  |  |  |  |
| **GRID** | **COLOR** | **VESSICLES (cm or mm scale; %age)** | **OTHER** | **SCAN 1** | **SCAN 2** | **SCAN 3** |
| A13 | Reddist surface, little bit of black | flat surface, stretched texture | cm gap between rock and gun | No match | No match | No match |
|  |  |  |  |  |  |  |
|  |  |  |  |  |  |  |
| B13 | Grey surface, some red | mm- to cm-scale; 50% |  | Clinop, 3 stars | Clinop, 3 stars | Clinop, 3 stars |
|  |  |  |  |  |  |  |
|  |  |  |  |  |  |  |
| C13 | Orange red surface wth dark patches | mm- to cm-scale; 50% |  | Clinop, 2 stars | Nontronite, 2 stars | No match found |
|  |  |  |  |  |  |  |
|  |  |  |  |  |  |  |
| D13 | lichen: skip |  |  |  |  |  |
|  |  |  |  |  |  |  |
|  |  |  |  |  |  |  |
| E13 | lichen: skip |  |  |  |  |  |
|  |  |  |  |  |  |  |
|  |  |  |  |  |  |  |
| F13 | Almost black, little brownish tint | stretched surface |  | No match found | Goetithe, 2 stars | No match found |
|  |  |  |  |  |  |  |
|  |  |  |  |  |  |  |
|  |  |  |  |  |  |  |
| **GRID** | **COLOR** | **VESSICLES (cm or mm scale; %age)** | **OTHER** | **SCAN 1** | **SCAN 2** | **SCAN 3** |
| A14 | Red surface | Highly vessiculated, mm- to cm-scale |  | Fe-Mont, 2 stars | No match | No Match |
|  |  |  |  |  |  |  |
|  |  |  |  |  |  |  |
| B14 | Orange red, with some black, tan color too | Rather smooth, slightly vessiculated, sub-mm scale, |  | Clinop, 3 stars | Clinop, 3 stars | Clinop, 2 stars |
|  |  |  |  |  |  |  |
|  |  |  |  |  |  |  |
| C14 | Orange red surface | mm- to cm-scale, 60% coverage | lichen all around it, only in one spot is it clea, which is where Alex shot | Hematite, 3 stars | Hematite, 3 stars | Hematite, 3 stars |
|  |  |  |  | Analcime, 3 stars | Analcime, 3 stars | Analcime, 3 stars |
|  |  |  |  | NH3_I/S, 2 stars and Dioptase 1 star | Dioptase 2 stars | NH3_I/S, 2 stars |
| D14 | lichen: skip |  |  |  |  |  |
|  |  |  |  |  |  |  |
|  |  |  |  |  |  |  |
| E14 | lichen: skip |  |  |  |  |  |
|  |  |  |  |  |  |  |
|  |  |  |  |  |  |  |
| F14 | lichen: skip |  |  |  |  |  |
|  |  |  |  |  |  |  |
|  |  |  |  |  |  |  |
| G14 | black shiny stretched out surface, lichen apparent, but shooting around it | stretched vessicles |  | NO match found | NO match found | NO match found |
|  |  |  |  |  |  |  |
|  |  |  |  |  |  |  |
|  |  |  |  |  |  |  |
| **GRID** | **COLOR** | **VESSICLES (cm or mm scale; %age)** | **OTHER** | **SCAN 1** | **SCAN 2** | **SCAN 3** |
| A15 | skipped |  |  |  |  |  |
|  |  |  |  |  |  |  |
|  |  |  |  |  |  |  |
| B15 | black orange red, with some dark patches | vessicular mm- to cm-scale; 70% | last scan was 0205 | hematite 3 stars | jarosite 3 stars | hematite 3 stars |
|  |  |  |  | jarosite 3 stars | nontronite, 2 stars | jarosite 3 stars |
|  |  |  |  | nontronite, 2 stars |  | nontronite, 1 star |
| C15 | Orange, black with some fresh glassy curface | mm- to cm-scale; 80% |  | jarosite 2 stars | jarosite 2 stars | jarosite 2 stars |
|  |  |  |  | Mont, 2 stars | Mont, 2 stars | nontronite, 1 star |
|  |  |  |  | Nontronite 1 star, Buddingtonite 1 star |  |  |
| D15 | lichen: skip |  |  |  |  |  |
|  |  |  |  |  |  |  |
|  |  |  |  |  |  |  |
| E15 | lichen: skip |  |  |  |  |  |
|  |  |  |  |  |  |  |
|  |  |  |  |  |  |  |
| F15 | black shiny surface | some vessicles, but mostly flat, little bit of orange |  | Goethite, 2 stars | No match | No match |
|  |  |  |  |  |  |  |
|  |  |  |  |  |  |  |
|  |  |  |  |  |  |  |
| **GRID** | **COLOR** | **VESSICLES (cm or mm scale; %age)** | **OTHER** | **SCAN 1** | **SCAN 2** | **SCAN 3** |
| A16 | lichen: skip |  |  |  |  |  |
|  |  |  |  |  |  |  |
|  |  |  |  |  |  |  |
| B16 | brown, red orange surface; looks like a hot altered surface | vessicular 70%, cm-scale; black inside vessicles |  | Phillipsite-Ca, 3 stars | Heulandite, 3 stars | Heulandite, 3 stars |
|  |  |  |  |  |  |  |
|  |  |  |  |  |  |  |
| C16 | Orange red, some grey in it, | dense with one mm-scale vessicle, |  | Jarosite, 3 stars | Jarosite, 3 stars | Jarosite, 3 stars |
|  |  |  |  |  |  |  |
|  |  |  |  |  |  |  |
| D16 | lichen: skip |  |  |  |  |  |
|  |  |  |  |  |  |  |
|  |  |  |  |  |  |  |
| E16 | lichen: skip |  |  |  |  |  |
|  |  |  |  |  |  |  |
|  |  |  |  |  |  |  |
| F16 | black shiny surface, stretched like snake skin; basically it is the top of the lobe as per above; some faint orange too |  |  | No match found | No match found | No match found |
|  |  |  |  |  |  |  |
|  |  |  |  |  |  |  |
|  |  |  |  |  |  |  |
| **GRID** | **COLOR** | **VESSICLES (cm or mm scale; %age)** | **OTHER** | **SCAN 1** | **SCAN 2** | **SCAN 3** |
| A17 | big hole, not possible:  skipped |  |  |  |  |  |
|  |  |  |  |  |  |  |
|  |  |  |  |  |  |  |
| B17 | Bright red brown, lichen in the bubbles; | 70-80% coverage, mm- to cm-scale | spectra isn't a flat line, so something else is there or it is the lichen that is showing up in the spectra | No match found | No match found | No match found |
|  |  |  |  |  |  |  |
|  |  |  |  |  |  |  |
| C17 | Orange and grey | 70% vessiculated, mm-scale, flat |  | Goethite 3 stars | Jarosite 3 stars | Jarosite 2 stars |
|  |  |  |  | Jarosite 3 stars | Mont 2 stars | Mont 2 stars |
|  |  |  |  | Mont 2 stars; Jarosite 1 star |  |  |
| D17 | lichen: skip |  |  |  |  |  |
|  |  |  |  |  |  |  |
|  |  |  |  |  |  |  |
| E17 | lichen: skip |  |  |  |  |  |
|  |  |  |  |  |  |  |
|  |  |  |  |  |  |  |
| F17 | black glassy surface, hint of orange |  |  | No match | No match | No match |
|  |  |  |  |  |  |  |
|  |  |  |  |  |  |  |
|  |  |  |  |  |  |  |
| **GRID** | **COLOR** | **VESSICLES (cm or mm scale; %age)** | **OTHER** | **SCAN 1** | **SCAN 2** | **SCAN 3** |
| A18 | Red, orange, tan and white all mixed together; bright | 70%, mm- and cm- scale | Very stable measurement, was sitting perfectly | Fe Smectite, 2 stars | Mont, 2 stars | Mont, 2 stars |
|  |  |  |  | Mg Illite 1 star |  | Dolomite, 1 star |
|  |  |  |  |  |  |  |
| B18 | Grey, tanned, slight orange | 80%, mm- cm-scale |  | No match | No match | Chrysocola, 2 stars |
|  |  |  |  |  |  |  |
|  |  |  |  |  |  |  |
| C18 | Grey, pale orange | Bright orange in the vessicles, 80% cm-scale |  | Jarosite 3 stars | Goethite 3 stars | Goethite 3 stars |
|  |  |  |  | Mont 2 stars | Jarosite 3 stars | Jarosite 3 stars |
|  |  |  |  |  |  |  |
| D18 | lichen: skip |  |  |  |  |  |
|  |  |  |  |  |  |  |
|  |  |  |  |  |  |  |
| E18 | Black glassy material on top just as before |  | end at 0241 | No match | No match | No match |
|  |  |  |  |  |  |  |
|  |  |  |  |  |  |  |
|  |  |  |  |  |  |  |
| **GRID** | **COLOR** | **VESSICLES (cm or mm scale; %age)** | **OTHER** | **SCAN 1** | **SCAN 2** | **SCAN 3** |
| A19 | tan color, light material | mm-cm-scale vesicles, 70% |  | No match found | No match found | No match found |
|  |  |  |  |  |  |  |
|  |  |  |  |  |  |  |
| B19 | tan color, llittle darker compared to previous spot | mm-cm-scale vesicles, 70% | 0.5 cm gap between gun and rock | Chrysocolla*** | Chrysocolla** | Chrysocolla** |
|  |  |  |  | Montmorillonite** | Montmorillonite** | Montmorillonite* |
|  |  |  |  | S_0245 | S_0246 | Buddingtontonight |
| C19 | brown glassy | cm-scale vesicles, 80-90% |  | No match found | No match found | No match found |
|  |  |  |  |  |  |  |
|  |  |  |  |  |  |  |
| D19 | brown glassy | cm-scale vesicles, 80-90% |  | No match found | No match found | No match found |
|  |  |  |  |  |  |  |
|  |  |  |  |  |  |  |
|  |  |  |  |  |  |  |
| **GRID** | **COLOR** | **VESSICLES (cm or mm scale; %age)** | **OTHER** | **SCAN 1** | **SCAN 2** | **SCAN 3** |
| A20 | Tan with grey | mm and cm scale vessicles, 70% |  | No match found | Goethite 2* | Goethite  2* |
|  |  |  |  |  |  |  |
|  |  |  |  |  |  |  |
| B20 | Same material |  | 10 cm up from last one | No match found | No match found | No match found |
|  |  |  |  |  |  |  |
|  |  |  |  |  |  |  |
| C20 | Rust red | high vessiculated material, mm scale, 90% | 10 cm up from last one | No match found | Goethite 3* | Goethite 2* |
|  |  |  |  |  | Jarosite 1* |  |
|  |  |  |  |  |  |  |
| D20 | Black, glassy, stretched out material | 60%, stretched out |  | No match found | No match found | No match found |
|  |  |  |  |  |  |  |
|  |  |  |  |  |  |  |
|  |  |  |  |  |  |  |
| **GRID** | **COLOR** | **VESSICLES (cm or mm scale; %age)** | **OTHER** | **SCAN 1** | **SCAN 2** | **SCAN 3** |
| A21 | Tan with grey and some black | mm and cm, 80% | Highly altered | Goethite 3* | Goethite 3* | Goethite 3* |
|  |  |  |  |  |  |  |
|  |  |  |  |  |  |  |
| B21 | Rust color with some grey | 2 giant 5cm across vessicles, and rest smaller in mm to cm scale, 80-90% | Highly altered | Goethite 3* | Goethite 3* | Goethite 3* |
|  |  |  |  |  |  |  |
|  |  |  |  |  |  |  |
| C21 | Rust color with grey and black | mm to cm scale, 90% | Highly altered;  a little bit of lichen but will shoot around it, Scan 0275 | Goethite 3* | Goethite 3* | Goethite 3* |
|  |  |  |  | Jarosite 1* | Jarosite 1* | Jarosite 1* |
|  |  |  |  |  |  |  |
| D21 | Black glassy, stretched out material | stretched out | stuff at the top | No match | No match | No match |
|  |  |  |  |  |  |  |
|  |  |  |  |  |  |  |
|  |  |  |  |  |  |  |
| **GRID** | **COLOR** | **VESSICLES (cm or mm scale; %age)** | **OTHER** | **SCAN 1** | **SCAN 2** | **SCAN 3** |
| A22 | Tan colored with some grey | 5-40 cm vessicles and then smaller ones mm to cm scale | trying to avoid giant vessicles when shooting it | Goethite 3* | Goethite 3* | Goethite 3* |
|  |  |  |  |  |  |  |
|  |  |  |  |  |  |  |
| B22 | Same as A22` | Same as A22 |  | Goethite 3* | Goethite 3* | Goethite 3* |
|  |  |  |  | Fe-smeceite 2* | Jarosite 2* | Fe-smeceite 2* |
|  |  |  |  | Gypsum 1*, Magnesium-illite 1* | Mont 1*, Ankerite 1* | Gypsum 1*, Magnesium-illite 1* |
| C22 | Browny rust color | mm, cm scale vessicles, 80-90% |  | No match | No match | Goethite 3* |
|  |  |  |  |  |  |  |
|  |  |  |  |  |  |  |
| D22 | Rust color | cm scale vessicles 60% | ` | No match | No match | Goethite 2* |
|  |  |  |  |  |  |  |
|  |  |  |  |  |  |  |
| E22 | black glassy material | stretched out | Reading at the top; scan number 0293 | No match | No match | No match |
|  |  |  |  |  |  |  |
|  |  |  |  |  |  |  |
