# Supplementary material for: Requirements for Portable Instrument Suites during Human Scientific Exploration of Mars
Source: Astrobiology. 2019 Mar 6;19(3):401–25. doi: 10.1089/ast.2018.1841 (PMC6442242; doi:10.1089/ast.2018.1841)
Supplement: Supplemental data [file Supp_Table1.zip › Supp_Table1.pdf]

Supplementary Table S1. Depiction of the Minerals Identified by the ASD for the Lobe in the Big Craters of the Moon National Monument and Preserve

A 10-cm grid was established, with 1–22 in the *x*-direction (from left to right), and A-I in the *y*-direction (from bottom to top of lobe), for example, the upper left corner of the lobe is I1 and the lower right corner of the lobe is A22. Information is only shown for locations where the lobe was physically present. Gray cells indicate that it was not possible to collect an ASD scan of that grid cell due to the presence of lichens, vegetation, or other reasons. This information is shown visually in Supplementary Figure S1. Data from scans with the highest level of confidence are included in Figure 9.
