# Supplementary material for: Requirements for Portable Instrument Suites during Human Scientific Exploration of Mars
Source: Astrobiology. 2019 Mar 6;19(3):401–25. doi: 10.1089/ast.2018.1841 (PMC6442242; doi:10.1089/ast.2018.1841)
Supplement: Supplemental data [file Supp_Fig1.pdf]

Supplementary Data

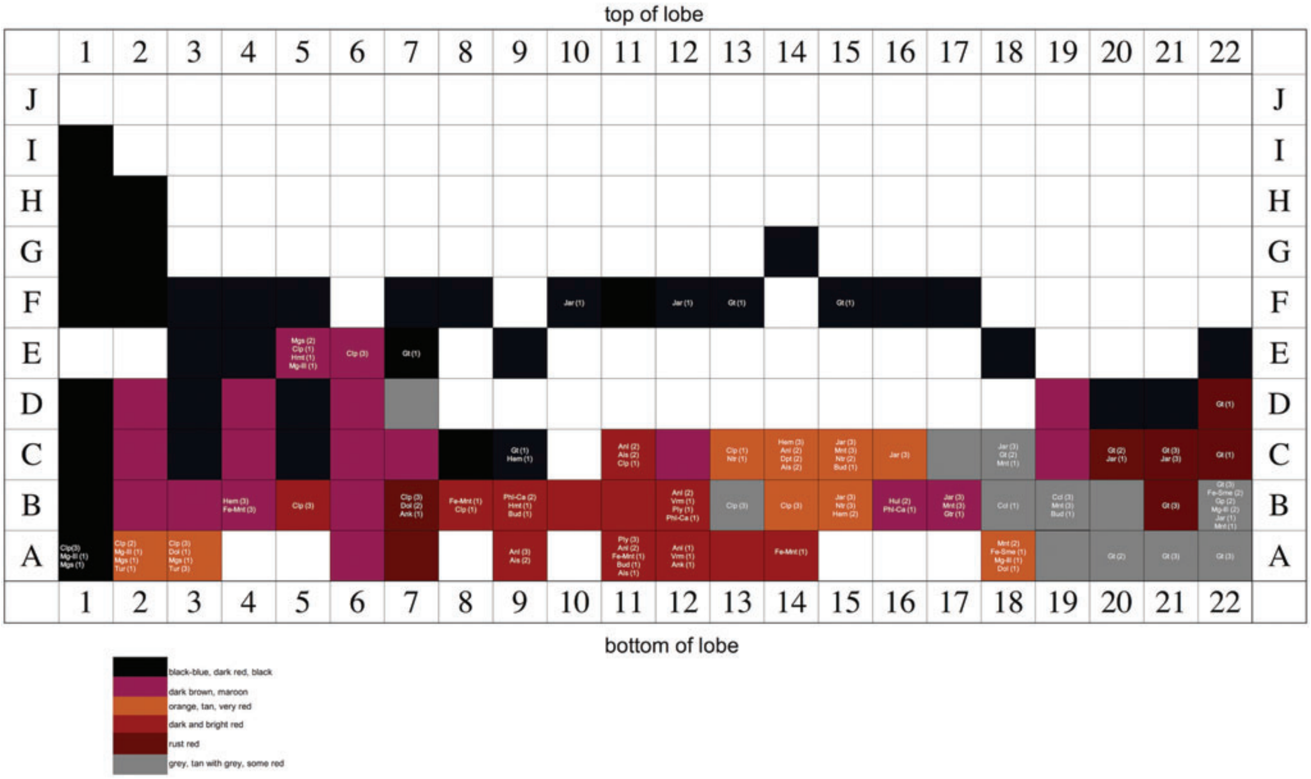

SUPPLEMENTARY FIG. S1. A visualization of the mineral identification data, found in Supplementary Table S1, from the outcrop assessed for basaltic alteration products. The outcrop was divided into a 10-cm grid across the surface of the north-facing side of the lobe. All mineral identifications are included here, and inclusion of a mineral in this figure does not reflect on the qualify or confidence of the identification; minerals with the highest level of confidence are included in Figure 9. Each spot was scanned a total of three times. The number in parenthesis is the number of times, out of 3, that a mineral was identified as such by the instrument. For example, ‘‘Hem (2)’’ means that from 2 out 3 three scans the instrument believes to have detected hematite. Mineral abbreviations are designated in Supplementary Table S2. Scans with ‘‘no match’’ found are not included.
