# Supplementary material for: Requirements for Portable Instrument Suites during Human Scientific Exploration of Mars
Source: Astrobiology. 2019 Mar 6;19(3):401–25. doi: 10.1089/ast.2018.1841 (PMC6442242; doi:10.1089/ast.2018.1841)
Supplement: Supplemental data [file Supp_Table2.pdf]

Supplementary Table S2. List of Mineral Abbreviations Used in Supplementary Table S1

Full Mineral Name	*Abbreviation*	*Note/Source*
Ammonio-illite/smectite (also, NH3-I/S)	Ais	2
Analcime	Anl	1
Ankerite	Ank	1
Buddingtonite	Bud	2
Chrysocolla	Ccl	1
Clinoptilolite	Clp	2
Dioptase	Dpt	3
Dolomite	Dol	1
Fe(Iron)-Montmorillonite	Fe-Mnt	1
Fe(Iron)-Smectite	Fe-Sme	1
Goethite	Gt	1
Gypsum	Gp	1
Harmotome	Hmt	3
Hematite	Hem	1
Heulandite	Hul	1
Jarosite	Jar	2
Magnesite	Mgs	1
Mg-Illite	Mg-Ill	1, 3
Montmorillonite	Mnt	1
Nontronite	Ntr	2
Palygorskite	Ply	2
Phillipsite with Calcium	Phl-Ca	3
Tourmaline	Tur	1
Vermiculite	Vrm	1

Notes on abbreviations: (1) As recommended by IUGS (www.bgs.ac.uk/scmr/docs/papers/paper_12.pdf), and consistent with USGS conventions (www.nwrc.usgs.gov/techrpt/sta16.pdf). (2) When a mineral was not listed in (1), we used the abbreviation as recommended for the USGS spectral library (https://speclab.cr.usgs.gov/PAPERS/paper.splib05a/review.6.2003/abbreviations.asc). Some of these overlap with other abbreviations from source (1). (3) Abbreviations for these could not be found, so we created these abbreviations.

For minerals with specific elemental abundances, a combination of approaches was used.
