# Supplementary material for: Requirements for Portable Instrument Suites during Human Scientific Exploration of Mars
Source: Astrobiology. 2019 Mar 6;19(3):401–25. doi: 10.1089/ast.2018.1841 (PMC6442242; doi:10.1089/ast.2018.1841)
Supplement: Supplemental data [file Supp_Fig2.pdf]

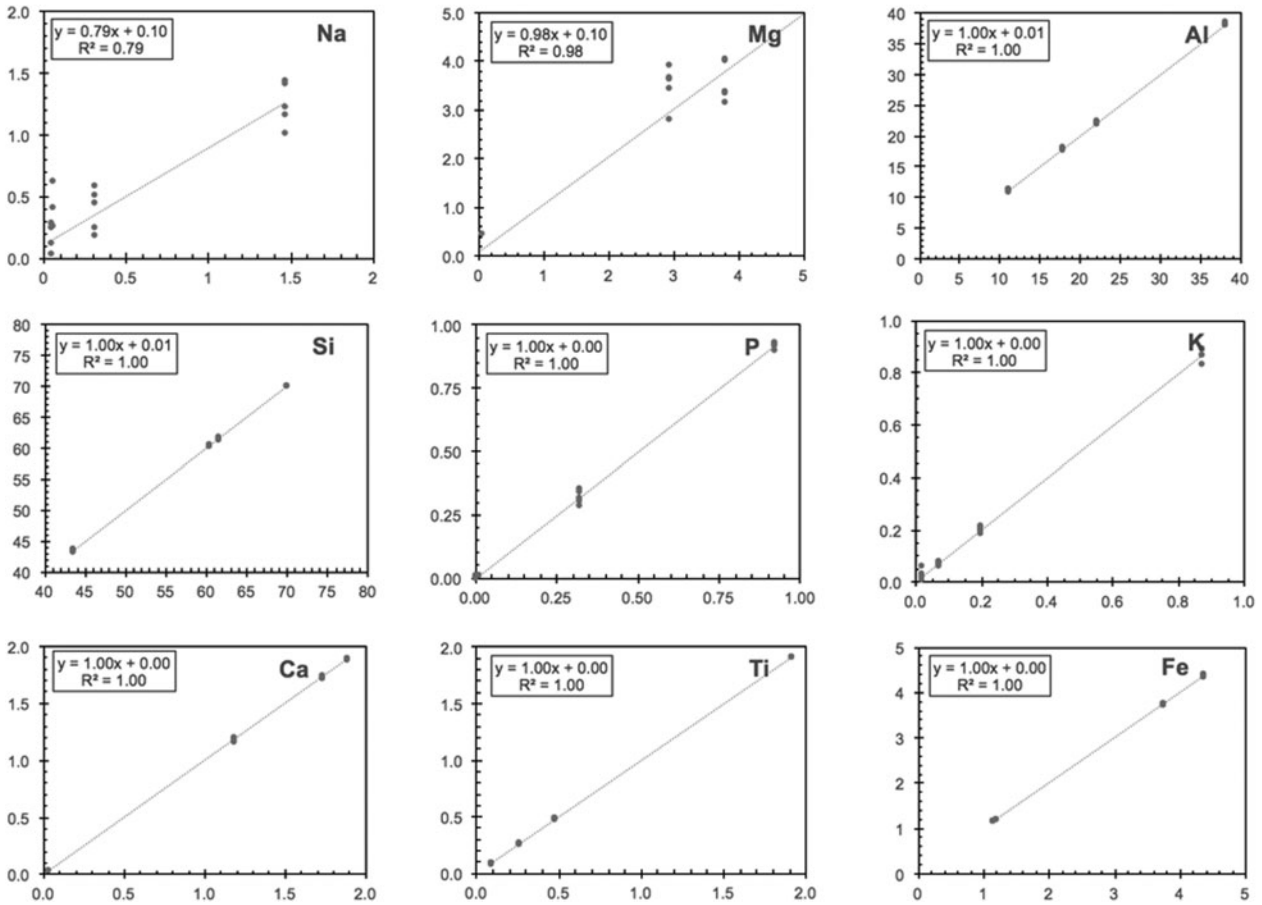

SUPPLEMENTARY FIG. S2. Mineral calibration for XRF. XRF calibration example for clay minerals (Illite and Montmorillonite), showing that individual calibrations (based on colors etc.) can be developed to collect more precise chemical data for specific mineral groups (e.g., previously identified by vis-NIR spectroscopy). vis-NIR, visible-near infrared; XRF, X-ray fluorescence.
